# The calcium channel proteins ORAI3 and STIM1 mediate TGF-β induced *Snai1* expression

**DOI:** 10.18632/oncotarget.25672

**Published:** 2018-06-29

**Authors:** Atrayee Bhattacharya, Janani Kumar, Kole Hermanson, Yuyang Sun, Humaira Qureshi, Danielle Perley, Adam Scheidegger, Brij B. Singh, Archana Dhasarathy

**Affiliations:** ^1^ Department of Biomedical Sciences, University of North Dakota School of Medicine and Health Sciences, Grand Forks, ND, USA; ^2^ Present address: MD Anderson Cancer Center, Mitchell Basic Sciences Research Building, TX, USA; ^3^ Present address: UT Health Science Center, San Antonio, San Antonio, TX, USA; ^4^ Present address: Habib University, University Avenue, Gulistan-e-Jauhar, Karachi, Pakistan

**Keywords:** calcium, TGF-β, SNAIL, EMT, ORAI3

## Abstract

Calcium influx into cells via plasma membrane protein channels is tightly regulated to maintain cellular homeostasis. Calcium channel proteins in the plasma membrane and endoplasmic reticulum have been linked to cancer, specifically during the epithelial-mesenchymal transition (EMT), a cell state transition process implicated in both cancer cell migration and drug resistance. The transcription factor SNAI1 (SNAIL) is upregulated during EMT and is responsible for gene expression changes associated with EMT, but the calcium channels required for *Snai1* expression remain unknown. In this study, we show that blocking store-operated calcium entry (SOCE) with 2-aminoethoxydiphenylborane (2APB) reduces cell migration but, paradoxically, increases the level of TGF-β dependent *Snai1* gene activation. We determined that this increased *Snai1* transcription involves signaling through the AKT pathway and subsequent binding of NF-κB (p65) at the *Snai1* promoter in response to TGF-β. We also demonstrated that the calcium channel protein ORAI3 and the stromal interaction molecule 1 (STIM1) are required for TGF-β dependent *Snai1* transcription. These results suggest that calcium channels differentially regulate cell migration and *Snai1* transcription, indicating that each of these steps could be targeted to ensure complete blockade of cancer progression.

## INTRODUCTION

The vast majority of cancer-associated deaths (about 90%) result from metastatic disease rather than the primary tumor. The spread of cancer cells from their primary regions of origin to distant metastatic sites is a multi-step process beginning with invasion of the cancer cells into surrounding tissue, intravasation of cells into the blood stream, extravasation to the secondary site, and finally, regrowth of the tumor cells as secondary metastases. A reversible process termed the ‘epithelial to mesenchymal transition’ (EMT) is an important developmental program that enables epithelial cells to lose apico-basal polarity, detach from their neighbors and from the extracellular matrix, and become more migratory and mesenchymal [reviewed in [1–3]]. This process is absolutely essential during the early developmental stages of gastrulation and neural crest migration [4, 5]. This developmental EMT program is hijacked by cancer cells to facilitate the process of metastasis, and can be induced by stimuli released in the tumor microenvironment such as the cytokine TGF-β [6]. TGF-β binds to the TGF-β receptor II (TGFBR2) in the plasma membrane and, through a well-studied signaling cascade [7–9], causes upregulation of *Snai1* gene expression [10].

The SNAI1 transcriptional repressor protein has been well studied in the context of EMT and is essential for gastrulation, as deletion of the gene results in lethality due to inhibition of embryonic development past the gastrula stage [4, 5]. SNAI1 is also positively correlated with metastatic tumors, and high levels of SNAI1 are predictive of decreased relapse-free survival in women with breast cancer [11]. Following binding to its cognate DNA sites, SNAI1 functions as a transcription factor, repressing expression of genes such as *E-cadherin* (*CDH1*) by recruiting chromatin remodeling complexes, leading to loss of cell-cell adhesion [12]. SNAI1 is also known to be upregulated in response to genotoxic stresses in the environment, thus preventing apoptosis [13]. A recent study suggested that while SNAI1-induced EMT is not absolutely needed for the physical migration of cells during metastasis, it does contribute to increased tumor survival and drug resistance [14].

TGF-β induced EMT in MCF7 breast cancer cells has also been shown to be associated with increased calcium influx into the cell [15]. Calcium levels in the cell can also influence cell migration during EMT [16–18]. Protein channels in the plasma membrane including the transient receptor potential (TRP) and ORAI channels function as store-operated calcium (SOC) channels that regulate the influx of calcium into the cell to modulate various biological processes. Importantly, TRPC1 and the Stromal Interaction Molecule 1 (STIM1) have been shown to facilitate cell migration during EMT [19]. Further, treatment with TGF-β caused an increase in calcium-induced calpain activity, which reduced E-cadherin protein levels, thereby increasing cell migration [19].

Blockade of SOCE was also shown to inhibit cell migration. For instance, pharmacological inhibition of SOCE with SKF [19] or silencing of *ORAI1* and *STIM1* was shown to inhibit cell migration in MDA-MB-231 breast cancer cells [20]. Further, chelation of intracellular calcium with BAPTA-AM reduced EGF-induction of cell migration in the MDA-MB-468 breast cancer cell line [16]. On the contrary, BAPTA-AM had opposite effects on two EMT transcription factors- it increased levels of TWIST1, but decreased the EGF- induced expression of *SNAI1,* a factor associated with decreased relapse-free survival in women with breast cancer [11]. This seemingly paradoxical finding can be potentially explained by a recent study suggesting that *SNAI1* is not absolutely needed for the physical migration of cells, but contributes to increased tumor survival and drug resistance [14]. Although these studies point to a link between calcium and migratory events leading to EMT, the identity of calcium channels needed for regulation of transcription factors that could modulate EMT was not explored.

**Figure 1:**
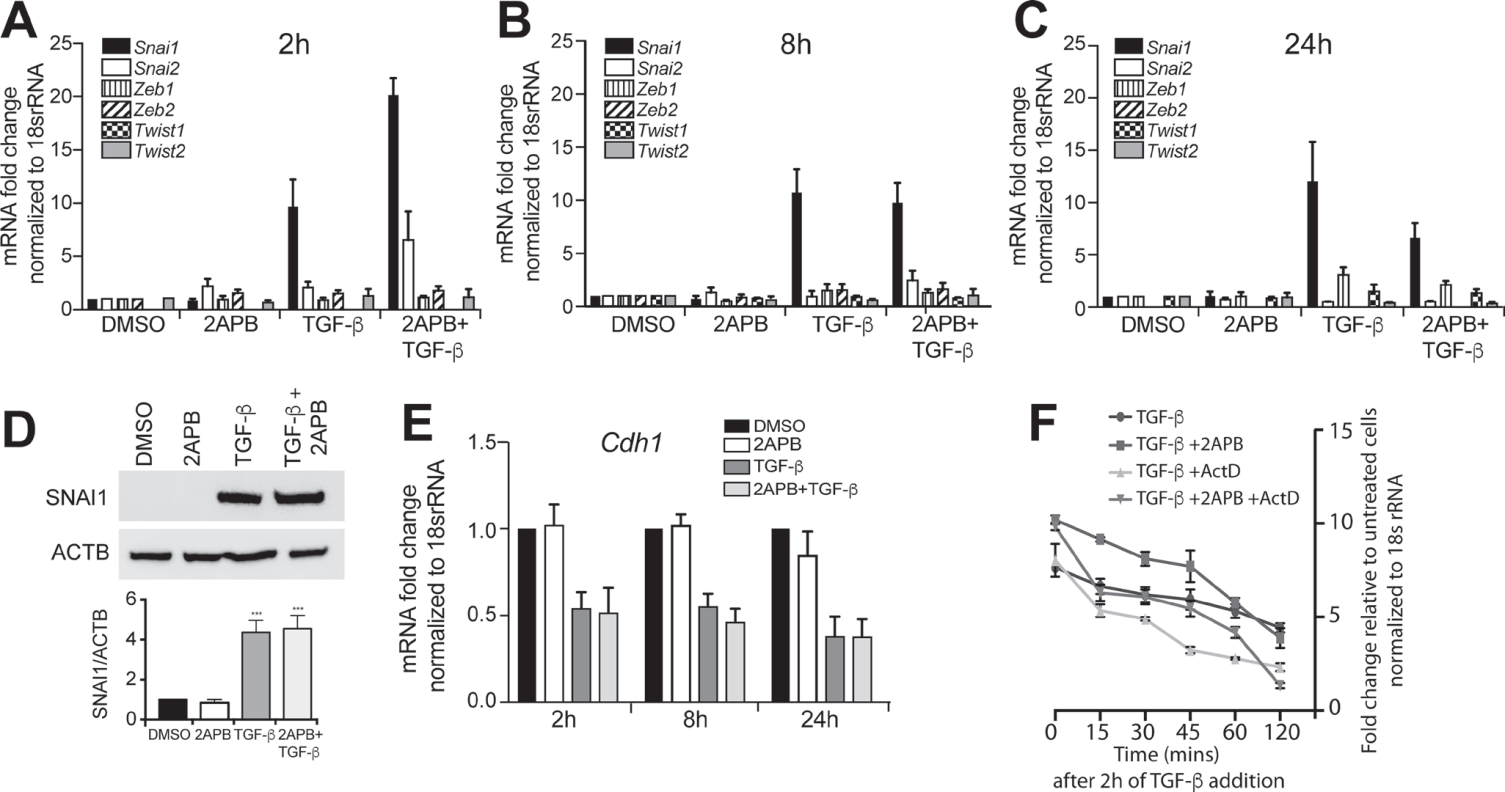
2APB amplifies the TGF-β dependent up-regulation of *Snai1* transcription NMuMG cells were serum-starved for 4 h, and then treated with DMSO or 2APB for 24 h, and TGF-β for 2 (**A**), 8 (**B**) and 24 (**C**) hours. RNA was isolated from NMuMG cells and cDNA prepared using reverse transcription. Expression of EMT genes was examined by real-time PCR of the cDNA using primers against each of the genes and normalized to *18S rRNA*. Data were derived from at least three independent biological replicates, and are represented as mean ± SEM values. The ^*^ indicates *p*-value of ≤ 0.05, and ^***^ indicates *p*-value ≤ 0.001 as measured by a paired *t*-test. (**D**) Protein analysis of NMuMG lysates treated with DMSO, or 2APB for 24 h, and with TGF-β for 8 h (added after 16 h of treatment with 2APB) as above was performed using western blotting with antibodies against SNAI1 and ACTIN. The blot is representative of at least 3 independent biological replicates. Quantitation was performed as described in methods, normalizing the signal to ACTB loading control. (**E**) Expression of E-cadherin, a downstream target of all the above EMT was measured from the same time points as in (A, B and C). (**F**) To test whether the increase seen in Snai1 expression is due to increase in transcription, cells were treated with DMSO or 2APB for 24 h, followed by TGF-β, and Actinomycin D was added 2 h after addition of TGF-β for 1 h. RNA isolation was followed in a time course of up to 2 h after Actinomycin D treatment. RNA was converted to cDNA and Snai1 expression measured as in (A, B, C and E). Statistical analyses were performed with Graphpad Prism software. ^*^= *p*-value ≤ 0.05.

Similar to our previous study [19], we noted that addition of the SOCE inhibitor 2-Aminoethoxydiphenylborane (2APB) prevented migration induced during EMT by TGF-β. However, 2APB amplified the TGF-β dependent expression of the *Snai1* gene, while induction of EMT genes *Zeb1, Zeb2, Twist1* and *Twist2* remained unaffected (Figure 1) at the time points tested. Expression of *Slug* (*Snai2*) was amplified relative to TGF-β alone in response to TGF-β+2APB at the 2 h point, but the effect was lost at later points. On the other hand, use of SKF96365 hydrochloride (SKF), another SOCE inhibitor that blocks SOC and voltage gated calcium channels, decreased the extent of TGF-β -induced *Snai1* induction (Supplementary Figure 1). To better understand how 2APB specifically increased TGF-β dependent *Snai1* expression, and to determine how calcium-signaling proteins alter cellular responses to TGF-β, we used RNA-sequencing to examine gene expression changes in the presence of 2APB. We observed that expression of a subset of genes in response to TGF-β was reversed with the addition of 2APB, which might reflect the reversion of the EMT phenotype. On the other hand, some *Snai1* target genes were either relatively unaffected, or affected to an increased level, suggesting that sustained *Snai1* expression could have downstream consequences. Next, we show here that the 2APB dependent amplification of the TGF-β induced *Snai1* gene activation occurs in part via the AKT and NF-κB signaling pathways. Finally, we show that 2APB appears to activate the ORAI3 [21–24] calcium channel, as knockdown of ORAI3 (or its interacting partner protein STIM1) results in loss of *Snai1* activation even in the presence of TGF-β. Taken together, these studies highlight the fact that cancer therapies should not only target physical migration of cells (EMT), but also prevent cancer cell survival and drug resistance through targeting genes like *SNAI1*, which are associated with increased chemoresistance.

## RESULTS

### 2APB amplifies the TGF-β dependent up-regulation of *SNAI1* transcription

We previously demonstrated that blocking calcium influx hindered EMT as seen by loss of cell migration [19]. Further, previous work has demonstrated that inhibition of SOCE could differentially affect transcription of EMT proteins [16]. However, the calcium channel essential for observed *Snai1* upregulation in response to blocking calcium entry has not yet been identified. To evaluate how SOCE influenced EMT transcription factor expression in response to TGF-β, we induced EMT in the murine mammary gland cell line, NMuMG. We found that addition of TGF-β up-regulated *Snai1* expression ~9-fold relative to DMSO treated cells within 2 hr of TGF-β treatment (Figure 1A), as expected. Upon treatment with both TGF-β and 50μM of the SOCE modulator 2APB (2- aminoethoxydiphenyl borate), there was a further increase in *Snai1* transcription (~20-fold total increase relative to DMSO) (Figure 1A). The increase in *Snai1* mRNA expression gradually decreased over time (within 24 hr). SNAI1 protein levels remained elevated with both TGF-β and TGF-β+ 2APB treatments relative to DMSO or 2APB controls as assessed by western blot (Figure 1D). Further, the expression of *Slug* (*Snai2*) was not significantly increased with TGF-β treatment at 2 h, but increased ~6 fold when cells are treated with 2APB and TGF-β in combination (Figure 1A). Transcription of the other EMT factors appear unaffected even after longer treatments with TGF-β at the time points tested (Figure 1A–1C). Together these results suggest that only *Snai1* gene expression is increased following TGF-β induction, and maintained above background in the presence of 2APB (Figure 1A–1C) at the time points tested.

As blockade of SOCE has been previously shown to prevent cellular migration associated with EMT [16, 19], we next asked if the SNAI1 protein was still functional. To this end, we assayed expression of E-cadherin mRNA, a well-known downstream target of SNAI1 (Figure 1E). Consistent with SNAI1 remaining fully functional, E-cadherin mRNA levels are down-regulated with TGF-β treatment, and remain low even with addition of 2APB (Figure 1E). Unlike 2APB, treatment of cells with SKF96365 hydrochloride (SKF), a SOCE inhibitor that blocks STIM1, TRPC, ORAI and voltage gated calcium channels, the TGF-β induced increase in *Snai1* mRNA expression was decreased, but not completely blocked by SKF (Supplementary Figure 1A). Use of SKF did not significantly influence expression of other tested EMT transcription factors (Supplementary Figure 1B). We also tested the effect of 2APB on *SNAI1* expression in the metastatic MDA-MB-231 breast cancer cell line (Supplementary Figure 2). We noted sustained increase in *SNAI1* expression at all time points tested above the levels seen with TGF-β alone. While *SNAI2* expression increases with TGF-β, there is no additional effect on its expression with 2APB treatment (Supplementary Figure 2), and other EMT factors are unaffected by 2APB treatment as well at the time-points and concentrations tested.

Next, to determine whether the increase in *Snai1* expression is due to elevation of transcription or, alternatively, reflects increased mRNA stability, we used Actinomycin D, a commonly used inhibitor of transcription. We induced *Snai1* gene expression for 2 h with TGF-β or TGF-β + 2APB, followed by Actinomycin D treatment (Figure 1F). *Snai1* mRNA expression is highest at 2 h following TGF-β addition and then gradually decreases over time, as previously observed. When cells are treated with Actinomycin D, there is a more rapid loss of *Snai1* mRNA expression relative to cells treated with TGF-β alone, suggesting that TGF-β addition affects *Snai1* gene transcription. With the addition of 2APB, we see a similar result to the TGF-β treatment, although the initial levels of *Snai1* mRNA are higher, there is still a gradual loss of transcription over time, as seen with the TGF-β treatment alone. Addition of Actinomycin D prior to the treatment with TGF-β and 2APB results in rapid loss of *Snai1* transcription, similar to the TGF-β with Actinomycin D treatment, suggesting that addition of 2APB does indeed affect transcription of *Snai1*. Together, these findings show that 2APB causes a specific increase in TGF-β- dependent transcription of *Snai1*, but not other EMT genes at the time points tested.

### Addition of 2APB reverses TGF-β -specific gene expression to inhibit migration

To obtain a genome-wide view of the gene expression changes induced by TGF-β then affected by 2APB, we performed RNA-sequencing on NMuMG cells that were treated with Dimethylsulfoxide (DMSO, vehicle control), TGF-β, or TGF-β+2APB for 24 h (Supplementary Figure 3A). Principal component analysis (PCA) plot (Supplementary Figure 4) generated from the RNA-seq data indicated that the replicates clustered together by treatment groups (DMSO, TGF-β and 2APB+TGF-β treatment groups), as expected. Differential expression analysis revealed that there were 5,992 genes significantly altered in TGF-β treatment relative to DMSO, and this number increased to 7,326 when 2APB was added to TGF-β (Supplementary Figure 3B). Of these 7,326 genes, 5,185 were also differentially expressed in response to TGF-β alone (Supplementary Figure 3B, 3C). Therefore, 2,141 genes were uniquely altered in expression due to 2APB+TGF-β alone. Of the genes that were upregulated in both comparisons (3,105 with TGF-β only and 3,671 with 2APB+TGF-β), there was a substantial overlap between the two (2,516 genes were common to both datasets). This left 1,155 genes that were unique to the 2APB+TGF-β dataset. Similarly, of the differentially downregulated genes (2,887 with TGF-β and 3,655 with 2APB+TGF-β), 2,606 genes overlapped in both datasets. We generated network maps and functional analyses of differentially expressed genes in all three comparisons (TGF-β vs DMSO, TGF-β+2APB vs DMSO, and TGF-β vs TGF-β+2APB) using QIAGEN's Ingenuity Pathway Analysis (IPA) software. All three comparisons generated the same top 5 significantly altered gene ontologies that are important for EMT, namely cell movement, cellular development, cell-to-cell signaling and interaction, cell growth and proliferation, and cell death and survival, although with different numbers of molecules in each category (Supplementary Figure 3D). Overall, 2APB and TGF-β treatment appears to result in a set of gene expression changes similar to the changes due to TGF-β alone. The full list and IPA analyses are available as Supplementary Files 1 and 2.

**Figure 2:**
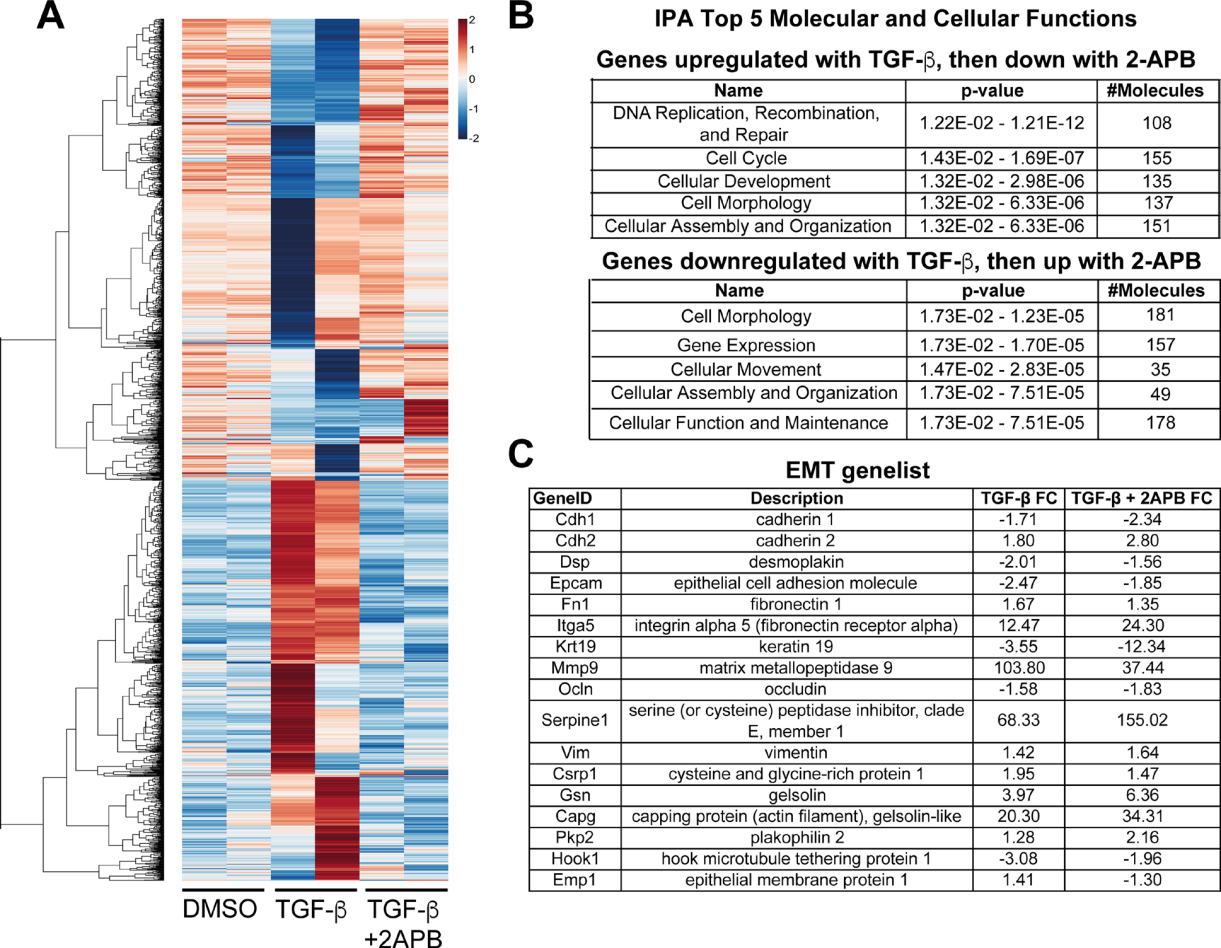
Reversal of gene expression comparing TGF-β to TGF-β+2APB treatments (**A**) RNA isolated from NMuMG cells treated with DMSO, TGF-β or TGF-β+2APB was sequenced, and gene expression changes calculated (see Supplementary Figure 1 and methods for details). Using this gene list, we extracted genes with expression changes when treated with TGF-β that were reversed by the addition of 2APB. We selected genes that had a range of ratio values of –1.5 to –0.5 and hierarchical clustering of their Pearson correlation values was performed using the aheatmap function in the R package NMF v0.20.6. (**B**) Genes that were upregulated with TGF-β addition, and then downregulated with 2APB addition were analyzed using IPA. The top five scoring hits in these categories using IPA are shown, together with significance scores (*p*-values) and the number of genes included in each class. A similar analysis was conducted with genes that were downregulated with TGF-β, but then reversed (up-regulated) with 2APB, and analyzed with IPA as above. (**C**) A subset of EMT-linked genes and their fold changes with TGF-β and TGF-β+2APB.

We and others previously showed that blocking SOCE leads to loss of the EMT phenotype [16, 19]. To understand the effects of 2APB in altering the TGF-β response, we investigated which genes had an increased expression with TGF-β, but were then decreased in response to 2APB (an “Up-Down” pattern). Similarly, we also looked for genes that had reduced expression in response to TGF-β but then increased with 2APB (“Down-Up” pattern). We reasoned that these reciprocal gene expression changes might be reflective of the reversion of the mesenchymal phenotype seen after TGF-β induction. We found 739 genes that showed the “Up-Down” pattern, and 853 genes in the “Down-Up” group (Figure 2A, 2B). Gene ontology (GO) analysis of both the Up-Down and Down-Up genes resulted in top 5 significantly altered gene categories very similar to that of the overall analysis, namely, DNA replication, recombination and repair, cell cycle, cellular development, cell morphology, and cellular assembly and organization (Figure 2B, top panel). Genes that were downregulated with TGF-β but then upregulated with 2APB addition, and vice versa, included many of the same categories as above (Figure 2B, lower panel). Of note in this category is *Klf4*, which acts as a transcriptional activator of epithelial genes and as a repressor of mesenchymal genes [25]. *Klf4* is downregulated around 2-fold by TGF-β addition, and is upregulated with 2APB and TGF-β (~4 fold) (Supplementary File 1). Several integrins and other cytoskeletal genes are downregulated with TGF-β but seem to be increased in expression with 2APB and TGF-β (Supplementary File 1). These categories are seen in the GO analysis of the changes caused by TGF-β alone, (Supplementary Figure 3D), but change in an opposite fashion.

Interestingly, we noted upregulation of EMT-promoting genes with 2APB and TGF-β addition, including *Osm* [26, 27], *Msi1* [28] and 2*610018G03Rik* (*Mst4*) [29]. On the other hand, one of the top ten genes that is upregulated with 2APB during TGF-β induced EMT was *Reln*, which was previously shown to prevent TGF-β induced migration [30]. This suggested a potential mechanism for 2APB-dependent reversal of TGF-β induced migration, and further supports a role for calcium signaling in EMT. Consistent with the increase in *Snai1* transcription and no loss of SNAI1 protein (Figure 1), TGF-β –dependent expression of *Snai1* target genes were maintained even with 2APB treatment. For example, *Snai1* target genes *Cdh1* [12] and *Krt19* [31] displayed a fold change of –1.7 and –3.55 respectively with TGF-β treatment, and decreased to –2.34 and –12.34 with TGF-β+ 2APB treatment (Figure 2C). This is consistent with our data that SNAI1 protein levels are unaffected with 2APB addition (Figure 1D).

### 2APB activates AKT signaling in response to TGF-β, resulting in increased NF-κB at the *Snai1* promoter

**Figure 3:**
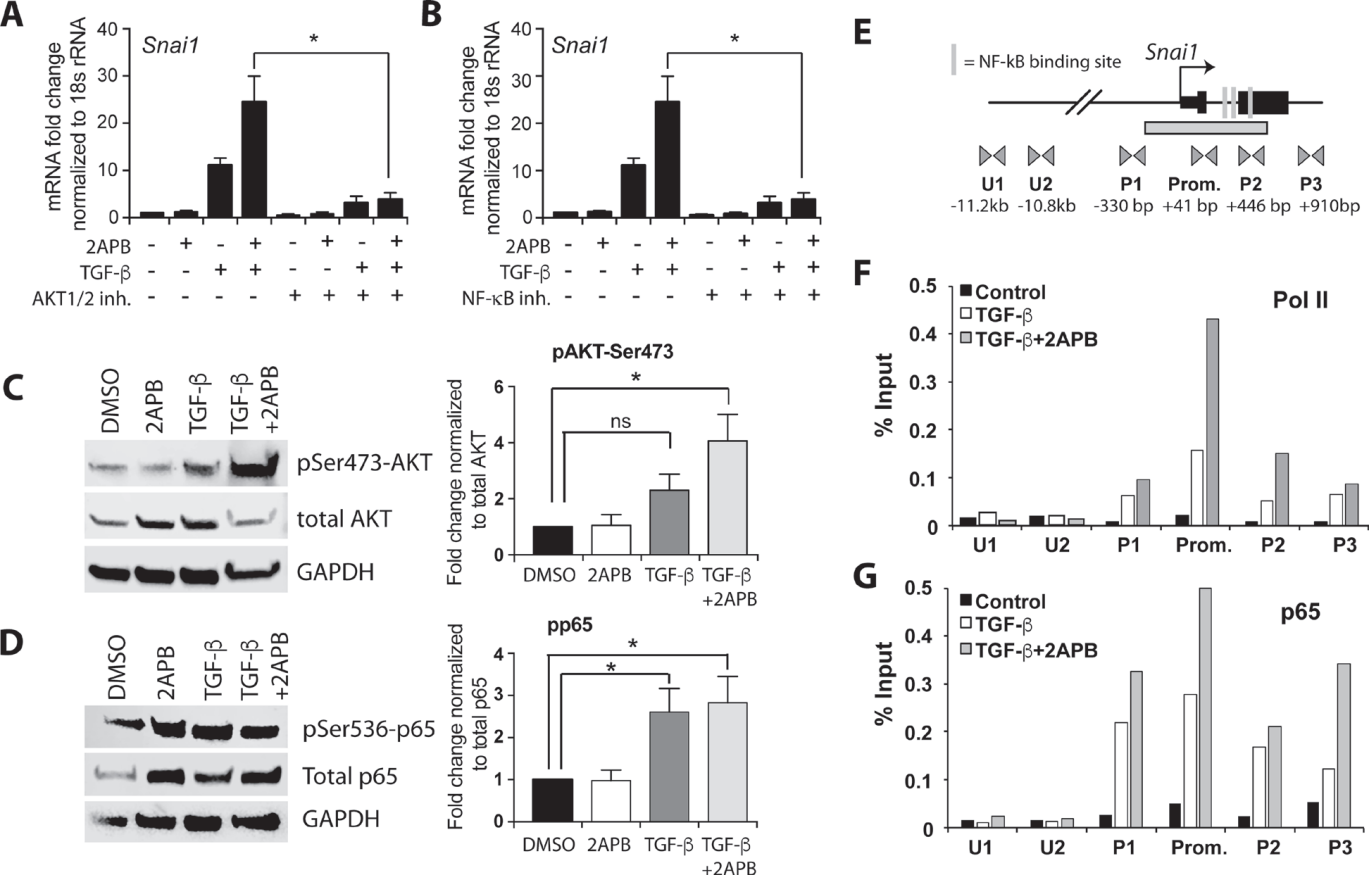
2APB increases TGF-β dependent activation of the AKT pathway and recruitment of NF-κB and Pol II to the *Snai1* promoter (**A**) NMuMG cells were serum starved for 4 hours and then treated with 2APB for 24 hours. At 18 hours, the cells were treated with 50 uM of the NFKB inhibitor ACHP for a 4-hour pretreatment before addition of TGF-β at 22 hours. (**B**) NMuMG cells were serum starved for 4 h and then treated with 10 uM of the inhibitor at 20 hours for a 2-hour pretreatment before the TGF-β treatment at 22 hours for 2 hours. (**C**) NMuMG cells were serum-starved for 4 h, and then treated with DMSO, 2APB, TGF-β or TGF-β+2APB for 24 h. Protein isolation from these cells followed by immunoblotting using antibodies to phospho- AKT^Ser473^, total AKT, and GAPDH. Data are representative of 3–4 independent biological replicates. Bar graphs next to the image represent the quantitation of blots using LiCOR image software, and statistical analyses performed using GraphPad Prism. ^*^= *p*-value ≤ 0.05. (**D**) The samples from (C) were immunoblotted for phospho-p65^Ser536^, total p65 (RelA subunit of NF-κΒ), and normalized to GAPDH as above. (**E**) Schematic representation of the primer sets used in chromatin IP covering ~3.8 Kb of the *Snai1* promoter region. The putative NF-κΒ binding sites are depicted as vertical lines. Locations of primer sets are indicated below the gene, as are the distances between the primer pairs in bp. (**F**) Chromatin immunoprecipitation (ChIP) was performed using antibodies against Pol II and p65, with IgG as a negative control. real-time PCR amplification of ChIP DNA across the *Snai1* locus reveals a peak of Pol II over the promoter region encompassed by primer set ‘Prom.’ in cells treated with TGF-β (white bars). This peak increases with 2APB treatment (grey bars). No discernable Pol II signal is noted in the vehicle (DMSO) treated cells (black bars). (**G**) While no p65 is apparent in DMSO treated cells, there is increased association of p65 at the *Snai1* promoter DNA with addition of TGF-β and with TGF-β+2APB. All data are representative of three independent biological replicates.

Comparison of differentially regulated gene networks between TGF-β and TGF-β+2APB treatments using IPA revealed significant changes in the AKT signaling pathway (Supplementary Figure 5). AKT signaling has been previously shown to be involved in both EMT [32, 33] and in regulation of *Snai1* expression through affecting the transactivation potential of NF-κB, including binding to the *Snai1* promoter and increasing its transcription [34–36]. Further, calcium is known to modulate AKT phosphorylation [37]. To test whether the AKT pathway was involved in the increase in *Snai1* transcription seen with 2APB and TGF-β addition, we treated NMuMG cells with Akti-1/2, a potent and selective dual Akt1 and Akt2 inhibitor [38]. There was little to no change in *Snai1* transcription both with Akti-1/2 with DMSO, or in combination with 2APB (Figure 3A). However, when used in combination with TGF-β treatment, or with 2APB+TGF-β, there was a significant decrease in *Snai1* expression (Figure 3A), suggesting that the AKT pathway is required for TGF-β dependent *Snai1* transcription. Similar results were observed when the cells were treated with the inhibitor ACHP [39], which interferes with the DNA binding ability of NF-κB (Figure 3B).

As the phosphorylated form of AKT is indicative of activity in these pathways, we examined levels of total and phosphorylated AKT using immunoblotting (Figure 3C). Specifically, we investigated a form of AKT phosphorylated at serine 473 (AKT^Ser473^) [40], and found increased levels upon TGF-β treatment, which were not diminished with the addition of 2APB (Figure 3C). Examination of our RNA-sequencing data did not reveal significant mRNA changes in Rictor or mTOR genes, which are known to phosphorylate AKT^Ser473^, nor was there a change in levels of total AKT, although it is quite possible that protein levels might be increased. Regardless, it seemed likely that the AKT^Ser473^ isoform is able to sustain and increase *SNAI1* transcription through downstream signaling, possibly through NF-κB transactivation as previously reported [35, 36, 40]. Further, IPA analysis listed RICTOR as one of the top 5 sets of upstream regulatory pathways in the genes that were upregulated specifically with 2APB+TGF-β alone (Supplementary File 2). Phosphorylation of the RelA (p65) subunit of NF-κB leads to the recruitment of transcriptional coactivators such as CBP/p300, and enhances NF-κB dependent transcription [41, 42]. To test whether 2APB addition changed protein levels of p65, we used antibodies specific to total and phosphorylated p65. While TGF-β induced a significant increase in phospho-p65 relative to total p65, we observed no significant loss or gain in p65 phosphorylation when comparing TGF-β to 2APB and TGF-β treatments (Figure 3D).

As the AKT pathway is actively involved in NF-κB regulation, specifically stimulating its transactivation potential in the nucleus [35, 36], we sought to determine whether increased expression of *Snai1* might be due to direct and increased binding of p65 at its promoter. Therefore, we performed chromatin immunoprecipitation (ChIP) of p65 and the RNA polymerase II (Pol II) enzyme [43, 44] using primers designed across the *Snai1* promoter, gene body, and in a region ~11kb upstream from the Snai1 transcription start site (Figure 3E). We found that relative to DMSO-treated cells, there is increased association of Pol II at the promoter region of *Snai1* at 2 h after addition of TGF-β (Figure 3F). The amount of Pol II at the *Snai1* promoter is further increased with the addition of 2APB relative to TGF-β alone, consistent with increased transcription from the *Snai1* promoter (Figure 1A). Next, the binding of p65 at the promoter is increased in cells treated with TGF-β, and even more so with addition of 2APB (Figure 3G), suggesting that p65 activation in response to 2APB is responsible for the increased *Snai1* transcription. Thus, the AKT and NF-κB pathway activation appears to be responsible for the increased activation of *Snai1* induced by TGF-β in the presence of 2APB.

### ORAI3 and STIM1 mediate TGF-β -induced *Snai1* expression

While our data suggested that AKT and NF-κB were involved in the amplified TGF-β dependent *Snai1* transcription in response to 2APB, how this signal is transmitted to the nucleus to regulate gene transcription was still unclear. While 2APB is a widely used SOCE inhibitor known to block calcium signaling through ORAI1 and ORAI2 channels, some studies have suggested that 2APB increases calcium entry through the closely related ORAI3 channel that is activated by its interaction with the stromal interaction molecule 1 (STIM1) [45, 46]. STIM1 and STIM2 are important components of SOCE and integral type I membrane proteins of the endoplasmic reticulum (ER). Further, we noted that SKF decreases the expression of TGF-β induced *Snai1* (Supplementary Figure 1), suggesting involvement of SOCE channels and STIM1.

**Figure 4:**
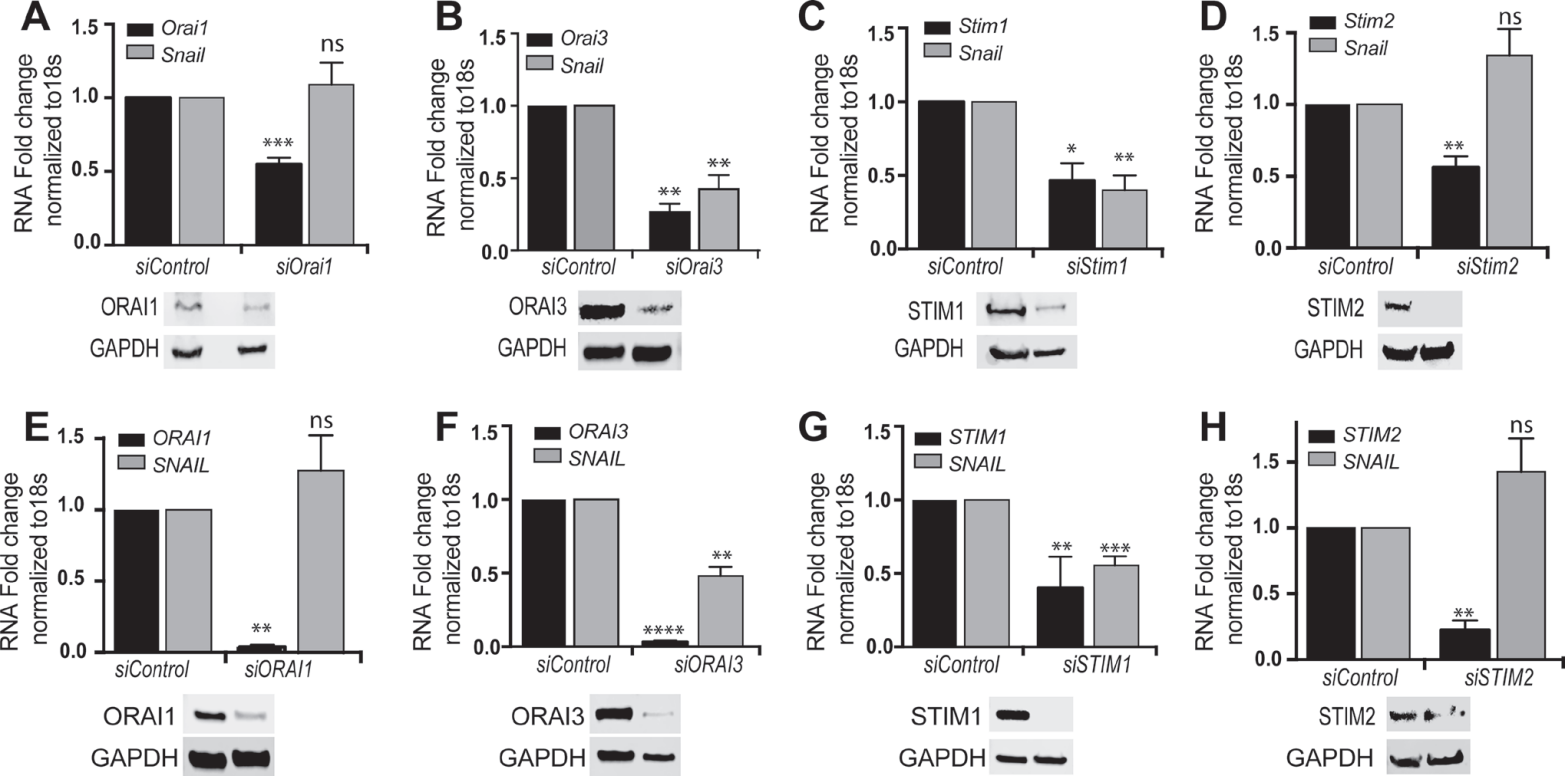
Orai3 and Stim1 silencing blocks TGF-β induced *SNAI1* transcription NMuMG (**A**–**D**) or MDA-MB-231 (**E**–**H**) cells were transfected with indicated *siRNAs* for 96 h using a final concentration of 100 picomoles of siRNA. The cells were treated with TGF-β for 2 h prior to RNA and protein isolation. RNA was converted to cDNA and RT-PCR performed to analyze both the gene knockdown efficiency for each gene, and Snai1 transcript levels. Western blots were performed against each protein to test efficiency of knockdown and normalized using GAPDH. All data are representative of at least 3 biological replicates (See Supplementary Figure 7 for quantitation). Statistical analyses were performed with Graphpad Prism software. ^*^= *p*-value ≤ 0.05, ^**^= *p*-value ≤ 0.01; ^***^= *p*-value ≤ 0.001.

To test whether calcium influx through ORAI and STIM proteins is required for *Snai1* expression, we knocked down *Orai1*, *Orai3, Stim1* and *Stim2* using siRNA in NMuMG (Figure 4A–4D) and MDA-MB-231 cells (Figure 4E–4H). We induced *Snai1* expression as before, through addition of TGF-β for 2 h. We then measured RNA and protein expression to validate knockdown (Figure 4). All 4 targets were significantly knocked down as seen by measurement of RNA (Figure 4A–4H, black bars) and protein (Supplementary Figure 7). We next measured if TGF-β induction of *Snai1* transcription was affected by the knockdowns relative to the siControl treatments. Of the four knockdowns tested, only loss of ORAI3 and STIM1 impact expression of *Snai1* in response to TGF-β treatment (Figure 4, gray bars), which is consistent with the results seen with 2APB and SKF treatments. TGF-β dependent *Snai1* transcription was maintained even with a partial knockdown of *Orai1* (Figure 4A) unlike with *Stim1* and *Orai3*. Further, we found no significant changes in any of the other EMT factors in response to knockdown of these channel proteins (Supplementary Figure 6), suggesting their specific influence on *Snai1* transcription.

### 2APB-dependent SOCE occurs through ORAI3

**Figure 5:**
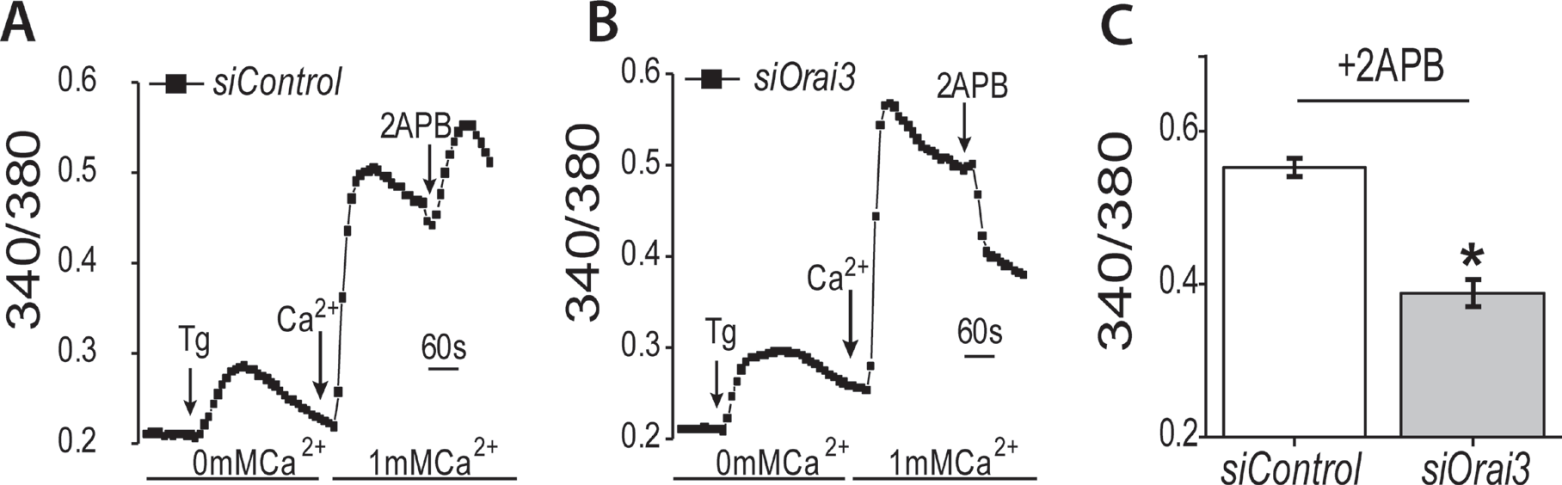
Orai3 silencing blocks 2APB dependent increase in SOCE Calcium imaging was performed in control (**A**) and *Orai3* knockdown (**B**) NMuMG cells. Analog plots of the fluorescence ratio (340/380) from an average of 40–60 cells are shown. (**C**) Quantification (mean ± SD) of fluorescence ratio (340/380). All data are representative of at least 3 biological replicates. Statistical analyses were performed with Graphpad Prism software. ^*^= *p*-value ≤ 0.05.

We next tested whether store depleted calcium influx that is altered with 2APB is further limited through knockdown of *Orai3*. We measured calcium levels using Fura-2 in *siControl* (Figure 5A) and si*Orai3* treated (Figure 5B) NMuMG cells. In the absence of external calcium (0 mM Ca^2+^), the addition of Thapsigargin (Tg, a sarcoendoplasmic reticulum calcium transport ATPase pump blocker, which thereby releases calcium from the internal ER stores), did not produce a significant difference between the control and *siOrai3* treated samples (Figure 5A and 5B). Initiation of calcium entry by the addition of 1mM calcium was not significantly different between the two sets of cells. However, addition of 2APB showed a statistically significant difference between the *siControl* and *siOrai3* treated cells (Figure 5A–5C), whereby there was a drop in calcium entry in cells that were treated with both 2APB and *siOrai3*. This loss of calcium influx with 2APB is about half of the level seen with the siControl treated cells (Figure 5C). This is consistent with the fact that knockdown of *Orai3* is not absolute (Figure 4B), and there is still some mRNA message in the cell. Taken together, these results support the idea that the influx of calcium through ORAI3 upon 2APB addition [45, 47] might be responsible for *Snai1* activation.

### Effect of *Orai3* silencing on migration and *Snai1* transcription in response to TGF-β

**Figure 6:**
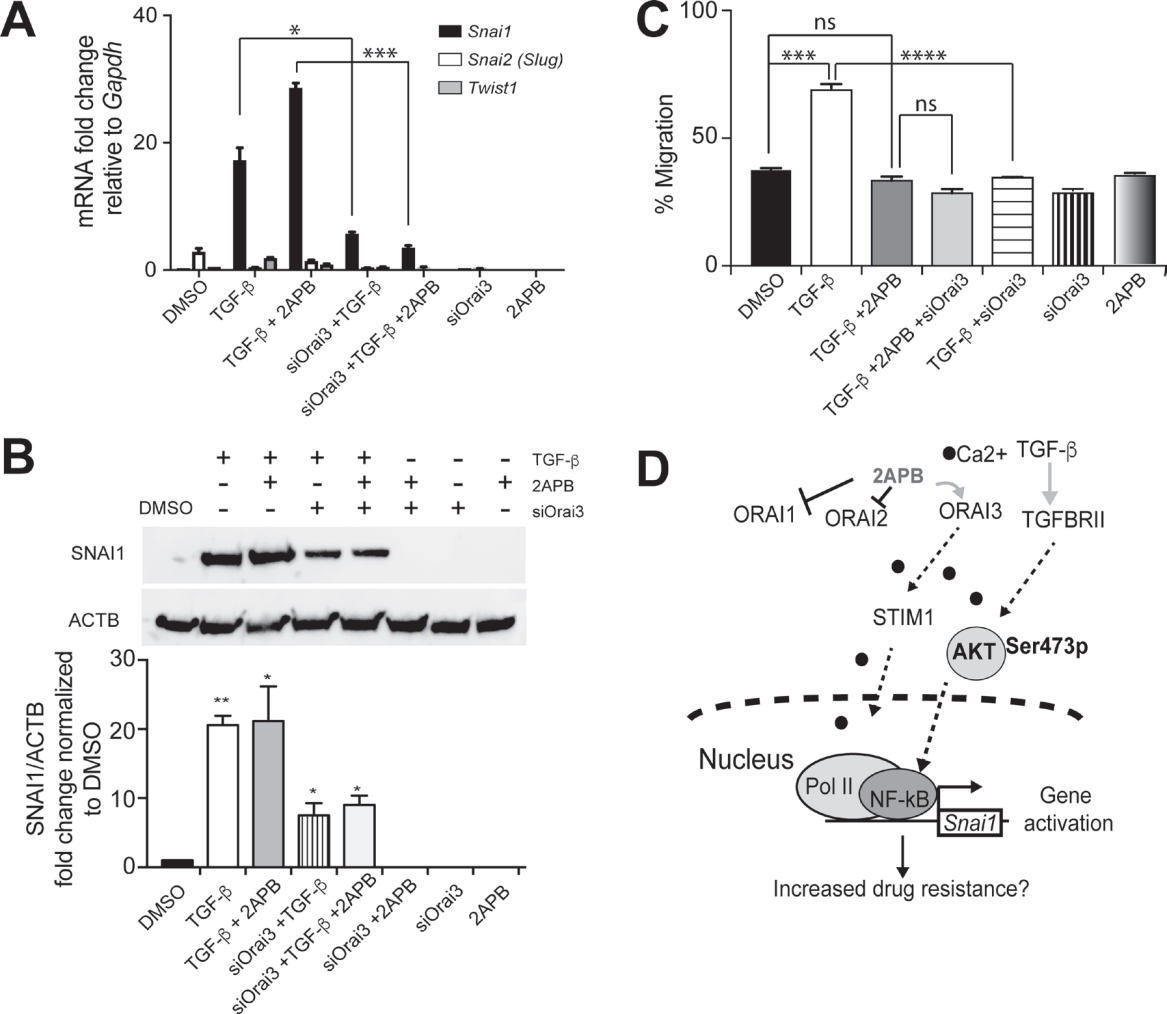
Orai3 silencing inhibits both cell migration and *Snai1* transcription in response to TGF-β (**A**) NMuMG cells were treated as indicated with TGF-β, TGF-β+2APB or DMSO, in the presence or absence of siOrai3 and RNA isolated. RNA was converted to cDNA and analyzed by real-time PCR using primers specific to mouse *Snai1, Snai2* or *Twist1,* and normalized to Gapdh. Data represent the average of 3 individual biological replicates. (**B**) Proteins isolated from the same cells as in (A) were evaluated for SNAI1 expression by immunoblotting. Antibody to ACTIN was used a loading control, and the blots are representative of at least 3 independent biological replicates. Blots were quantitated using the LiCOR imaging software and are represented as SNAI1/ACTB signal, after normalizing to DMSO control. Error bars represent SEM and statistical analyses were performed Graphpad PRISM. ^*^= *p*-value ≤ 0.05, ^**^= *p*-value ≤ 0.01 relative to control. (**C**) Confluent NMuMG cells in a 6-well plate were serum starved for 4 h prior to treatment, and TGF-β (for 8 h) and/or 2APB (for 24 h) were added to the wells prior to wounding using a sterile 200 ul tip. Three representative fields were marked and imaged immediately at time of (0 h) and a time period after (8 h) wounding as described in materials and methods. The images were captured using an Olympus IX71 microscope camera. All data are representative of at least 3 biological replicates. Statistical analyses were performed with Graphpad Prism software. ^*^= *p*-value ≤ 0.05, ^**^= *p*-value ≤ 0.01; ^***^= *p*-value ≤ 0.001; ^****^= *p*-value ≤ 0.0001. (**D**) Model for ORAI3-mediated *Snai1* upregulation. AKT (green oval) pathway can be activated by both calcium (black circles) and by TGF-β signaling. 2APB prevents SOCE via ORAI1 and ORAI2, while increasing calcium influx through ORAI3. Activation of AKT triggers increased binding of p65 at the *Snai1* promoter, leading to increased recruitment of Pol II and hence transcription of *Snai1*.

We next focused on delineating the specific role of ORAI3 in migration versus *Snai1* transcription in response to TGF-β. Consistent with the idea that ORAI3 channel protein is required for *Snai1* expression, a significant decrease (around 8 fold) in *Snai1* expression was observed when *siOrai3* treated cells were induced with TGF-β and 2APB, relative to TGF-β and 2APB (Figure 6A). Interestingly, knockdown of *Orai3* also caused a significant loss of TGF-β induced *Snai1* transcription relative to TGF-β alone, even without addition of 2APB (Figures 4B, 4F and 6A). This result suggested that ORAI3 channel is needed for robust activation of *Snai1* expression. Knockdown of *Orai3* also repressed the TGF-β dependent induction of *Snai2*, similar to 2APB (Figure 6A). We also noted that the loss of *Snai1* expression seen as a result of *siOrai3* treatment was reflected as a loss of SNAI1 protein expression (Figure 6B). While we do not observe a complete loss of SNAI1 protein expression, this is to be expected, as we still observed a small amount of protein by western blot when we knocked down *Orai3* (Figure 4B).

Having established that ORAI3 allows calcium entry and is primarily needed for increased *Snai1* expression, we asked if ORAI3 was also needed for cell migration that occurs as a result of TGF-β induced EMT. Therefore, we tested wound healing by a scratch assay in cells following induction with TGF-β (Figure 6C). As expected, cells treated with TGF-β resulted in more rapid migration and wound healing than DMSO-treated controls, or cells treated with 2APB alone (Figure 6C). We have previously demonstrated that inhibition of SOCE with SKF reduces cell migration caused by TGF-β [19], and we see the same with addition of 2APB. Moreover, we did not see a significant difference (Figure 6C) in wound closure relative to vehicle treated cells when we compared TGF-β induced cells that were also treated with 2APB or *siOrai3,* or both, indicating that 2APB and *siOrai3* both had similar effects in hindering cell migration induced by TGF-β. Together, these results suggest that while 2APB hinders cell migration, it is essential for modulation of *Snai1* expression. ORAI3 is thus a critical ion channel that is needed for *Snai1* expression, and knockdown of *Orai3* is capable of inducing both loss of cell migration and *Snai1* expression in response to TGF-β.

## DISCUSSION

Blockade of SOCE has been demonstrated to influence cell biological processes, including the cell migratory phenotype associated with EMT, making calcium channels an attractive target for chemotherapy. Here, we sought to understand how 2APB could specifically amplify the TGF-β induced *Snai1* expression levels. To do this, we utilized RNA-sequencing to obtain a global view of genes that were differentially regulated with TGF-β in the presence or absence of 2APB. Our analyses revealed substantial overlap between TGF-β and TGF-β+2APB datasets, suggesting that 2APB maintained the TGF-β dependent regulation of genes to a large extent (Supplementary Figure 3). However, TGF-β dependent changes in expression of a subset of genes that facilitate cell-extracellular matrix adhesion such as integrins, were reversed by addition of 2APB (Figure 2A). This explained how blocking SOCE could affect the migration phenotype seen with EMT. However, EMT-related genes including *Snai1* were upregulated upon TGF-β induction, and maintained or increased their relative expression even with the addition of 2APB. Expression of *Snai1* target genes including *Krt19*, *Ocln*, *Dsp*, etc. was also maintained even with 2APB treatment (Figure 2C). These observations are consistent with previously published data that *Snai1* upregulation and the migratory phenotype seen with TGF-β addition can be physically uncoupled [43].

Pathway analysis of the RNA-seq data revealed that the AKT network was differentially regulated between TGF-β and TGF-β+2APB (Supplementary Figure 5 and Supplementary File 2). Cytosolic AKT signaling has been shown previously to trigger a signaling cascade ultimately affecting the transactivation potential of NF-κB subunit p65 in the nucleus through a region located upstream of the *Snai1* promoter [35, 36]. AKT and p65 were therefore potential candidates for the observed increase in *Snai1* transcription. We tested this idea in two ways. First, treatment with inhibitors targeting either AKT1/2 (Akti-1/2) or that prevented p65 binding to DNA (ACHP) both resulted in loss of TGF-β dependent *Snai1* transcription (Figure 3A and 3B). *Snai1* transcription was also abolished in response to both TGF-β and TGF-β+2APB. We also noted increased phosphorylation of AKT^Ser473^ in response to TGF-β and TGF-β+2APB relative to control treated cells, suggesting activation of AKT signaling even with 2APB addition. Secondly, we showed direct association of p65 and RNA Polymerase II at the *Snai1* gene promoter (Figure 3E–3G) with TGF-β treatment. We further demonstrated an increase in both RNA Polymerase and p65 with addition of 2APB and TGF-β, which is consistent with the increased levels of *Snai1* transcript relative to TGF-β alone. The increased phosphorylation status of AKT in response to both TGF-β and TGF-β+2APB has consequences for both cell growth and metastasis (30–33). Further, both AKT (34,35) and its downstream target NF-κB (36,37) have been previously linked to an increase in drug resistance. Activation of both AKT and NF-κB would result in increased *Snai1* expression, which in turn has been shown by several groups to confer drug resistance to cancer cells [14, 48–50]. Our RNA-seq data support this idea, as several known drug resistance genes (e.g. *Abcg1*, *Ercc1*, *Igfbp4*, and *Cyp1b1*) were upregulated in the 2APB dataset, but not with TGF-β alone. Additional studies are needed to determine whether calcium blockers that are proposed for use in clinical treatments might contribute to acquisition of drug resistance.

Secondly, we asked which calcium channel proteins were involved specifically in TGF-β induced *Snai1* transcription. 2APB was previously shown to block calcium entry through ORAI1 and ORAI2 channels [46]. We demonstrated that 2APB treatment increased calcium influx through the ORAI3 channel as suggested previously [45, 47], which was lost when ORAI3 was knocked down (Figure 5). The stromal interaction partners of ORAI channels, namely STIM1 and STIM2, were previously shown to facilitate TGF-β induced EMT, although it was suggested that STIM2 appears to participate in non-SOCE mechanisms [51]. Knockdown of these four channel proteins showed that loss of ORAI3 and STIM1, but not ORAI1 or STIM2, also reduced TGF-β dependent *Snai1* transcription (Figure 4). While we do not observe a complete loss of SNAI1 protein expression upon ORAI3 knockdown, this is to be expected due to incomplete knockdown of *Orai3* (Figure 4B), or possible compensation through ORAI1/2. Interestingly, both 2APB (which activates ORAI3) and *siOrai3* treated cells showed reduced TGF-β induced migration. We predict that first, in the presence of 2APB, some of the genes induced by TGF-β that contribute to cell migration are reversibly regulated through as yet unknown means, which reverts them to the non-migratory phenotype, even in the presence of active ORAI3 and SNAI1 proteins. Alternatively, changes at the level of proteins in response to 2APB+TGF-β (whether through stability, post-translational modifications, etc.) might play a role in the reversal of phenotype, but this remains untested. When *Orai3* is downregulated in the presence of TGF-β, loss of *Snai1* expression might prevent EMT gene expression programs from fully activating. These results further highlight the separation of cell migration from gene expression programs during EMT, and underscore the need to target both pathways.

On the other hand, ORAI1, which is inhibited by 2APB [46], does not appear to be as critical for *Snai1* expression as ORAI3. In support of this idea, knockdown of ORAI1, although partial as observed by western blots (Figure 4A and 4E and Supplementary Figure 7) did not result in any loss of TGF-β dependent *Snai1* transcription (Figure 4A and 4E). Taken together, our data suggest that calcium influx through ORAI3 and STIM1, and downstream signaling, is required for TGF-β dependent *Snai1* activation. No significant difference in wound closure was noted when comparing *Orai3* knockdown to 2APB treatment during TGF-β induced EMT suggesting that blocking multiple channels through 2APB or simply knocking down *Orai3* have similar effects in preventing cell migration during EMT. Crucially, contrary to 2APB+ TGF-β treatment, knockdown of *Orai3* when combined with TGF-β induction of EMT decreased *Snai1* expression while also reducing the migratory phenotype. This reinforces the importance of the ORAI3 and the ERα protein STIM1 in upregulation of *Snai1* gene expression. We can therefore predict a model whereby 2APB blocks calcium entry through ORAI1 and ORAI2, but allows calcium “leakage” [47] into the endoplasmic reticulum through ORAI3 in partnership with STIM1 (Figure 6D). In parallel, the calcium entry activates AKT [37] signaling, potentially through PI3K [52]. AKT in turn transactivates NF-κB subunit p65, which binds to the *Snai1* promoter, recruits RNA Polymerase and increases activation in response to TGF-β (Figure 6D).

The expression of ORAI proteins, and hence their functional role, appears to be highly dependent on the breast cancer subtype. For instance, *Orai1* appears to be upregulated in the poor prognosis basal breast cancer molecular subtype [53], and silencing of ORAI1 reduces the proliferation of breast cancer cell lines *in vitro* [53, 54] and *in vivo* [54], the invasiveness of MDA-MB-231 breast cancer cells *in vitro*, and metastasis *in vivo* [20]. The basal subtype breast cancers are also more likely to have higher mRNA levels of the canonical ORAI channel activator STIM1, and lower levels of its related isoform STIM2 [53]. However, SOCE in less aggressive, estrogen receptor positive luminal subtype of breast cancer cells occurs mainly through ORAI3 [24], and upon loss of estrogen receptor alpha, metastatic cells appear to switch to SOCE through the ORAI1 channel, [23, 24]. Finally, the importance of ORAI3 in conferring chemoresistance has been underscored by a recent study that linked ORAI3 overexpression and chemoresistance in human breast cancer data sets [55], and further demonstrated that ORAI3 overexpression conferred chemoresistance properties to cells. In summary, understanding individual roles of ORAI channel proteins in cancer proliferation, metastasis and chemoresistance has implications in development of targeted therapies to treat cancer. Future studies will address how these calcium channel proteins function during breast tumor progression and affect drug resistance and EMT. Additionally, the role of transiently expressed SNAI1, and the temporal expression of the other EMT transcription factors during EMT, their interplay with calcium channels, and especially their effect on the multistep process of metastasis formation is of continuing interest to our laboratories, and is the subject of ongoing research.

## MATERIALS AND METHODS

### Cell culture

NMuMG (ATCC #CRL-1636) and MDA-MB-231 (ATCC #HTB-26) cells were obtained directly from American Type Culture Collection, and were cultured at 37° C under 5% CO_2_ in DMEM/F-12 media (Thermo Fisher) containing 10% fetal bovine serum (FBS, Atlanta Biologicals). Cells were serum-starved for 4 h prior to treatment with TGF-β (Sigma # H8541).

### Treatments

Cells were treated with TGF-β (5 ng/ml final; Sigma-Aldrich) for 8 h prior to protein isolation, and for 2 h, 8 h or 24 h prior to RNA isolation, unless noted otherwise. Cells were treated with 2APB (50 uM final; Sigma-Aldrich) for a period of 24 h prior to TGF-β treatment. Actinomycin D (1 ug/ml final; Sigma-Aldrich) treatments were for 1 h after stimulation with TGF-β. Cells were treated with 10 uM final SKF96365 hydrochloride (Sigma, #567310-M) for a period of 24 h prior to TGF-β treatment for 2 h. For treatment with p65 inhibitor ACHP, NMuMG cells were serum starved for 4 hours. After serum starvation, the cells were treated with 2APB for 24 hours. At 18 hours, the cells were treated with 50 uM of the NFKB inhibitor ACHP for a 4-hour pretreatment as previously published [39] before addition of TGF-β at 22 hours. For the AKT1/2 inhibitor, NMuMG cells were treated with 10 uM of the inhibitor at 20 hours for a 2-hour pretreatment as described previously [38] before the TGF-β treatment at 22 hours for 2 hours.

### Transfection

Transfections of cells with *siRNAs* (mouse for NMuMG and human for MDA-MB231, Ambion) were performed using Lipofectamine 3000 for two rounds of transfection (50 pmol reverse for 48 h, 50 pmol forward for 48 h) using a final concentration of 100 pico moles of siRNA. The cells were treated with 2APB or TGF-β after 96 h of transfection for appropriate time points prior to RNA and Protein isolation.

### RNA isolation

RNA was isolated from cells using the RNeasy kit (Qiagen) according to the manufacturer's instructions. Genomic DNA was removed by on-column DNA digestion with RNase-Free DNase Set (Qiagen). RNA quality and concentration was assessed using a spectrophotometer (NanoDrop), and by electrophoresis on a 2% agarose gel.

### qRT-PCR

Total RNA was extracted from cells using RNeasy kit (Qiagen) and checked for integrity using agarose gel electrophoresis. One microgram of RNA was used to synthesize cDNA using random hexamer priming and SSRT-III reverse transcriptase (Life Technologies), followed by qPCR using Quantitect (Qiagen) primer assays or primers designed and ordered from IDT (see Supplementary Table 1). Data were normalized against *Rrn18S* gene transcripts (Quantitect, Qiagen). Data were derived from at least three independent biological replicates, and are represented as mean ± SEM values. Data were analyzed using the delta-delta Ct method as described previously [43, 56]. Statistical analyses were performed using the GraphPad Prism software, version 7.0.

### Library construction and RNA-sequencing

The total RNA isolated as described above was used for 50 bp single-end RNA-Sequencing at the University of Minnesota Genomics Center (UMGC) on the Illumina HiSeq 2000. RNA quality was assessed with the Agilent Bioanalyzer, and samples with high RNA integrity number (RIN > 8) were used for library construction following the manufacturer's (Illumina) instructions. In summary, 1 microgram of total RNA was oligo-dT purified using oligo-dT coated magnetic beads, chemically fragmented and then reverse transcribed into cDNA. The cDNA was fragmented, blunt-ended, and ligated to indexed (barcoded) adaptors and amplified using 15 cycles of PCR. Final library size distribution was validated using capillary electrophoresis and quantified using fluorimetry (PicoGreen) and via Q-PCR. Indexed libraries were normalized, pooled and then size selected to 320 bp ± 5% using Caliper's XT instrument. TruSeq libraries were hybridized to a single end flow cell and individual fragments clonally amplified by bridge amplification on the Illumina cBot. Once clustering was complete, the flow cell was loaded on the HiSeq 2000 and sequenced using Illumina's SBS chemistry. Two biological replicates for each treatment were sequenced, resulting in an average of 50 million reads per sample. Base call (.bcl) files for each cycle of sequencing were generated by Illumina Real Time Analysis (RTA) software. The base call files and run folders were then exported to servers maintained at the Minnesota Supercomputing Institute. Primary analysis and de-multiplexing were performed using Illumina's CASAVA software 1.8.2. The end result of the CASAVA workflow is de-multiplexed FASTQ files that were subject to subsequent analyses as described below.

### RNA-seq data analyses

Preliminary quality control analysis of fastq files was performed with FastQC v0.11.2 [57]. Reads were aligned to human genome (hg19), using TopHat v2.0.13 [58]. Read counts were summarized at the gene level using the featureCounts [59] function in the Rsubread v1.16.1 package [60]. Differential expression analysis was performed using the R/Bioconductor package DESeq2 v1.6.3 [61]. Genes were considered differentially expressed if they had a FDR of 0.05 or less and a mean count of 20 or more. Hierarchical clustering of Pearson correlation values was performed using the aheatmap function in the R package NMF v0.20.6 [62]. Venn diagrams were drawn with the R package VennDiagram v1.6.17 [63]. Network mapping and functional analyses were generated through the use of IPA (QIAGEN Inc., https://www.qiagenbioinformatics.com/products/ingenuity-pathway-analysis) [64]. RNA fastq files are accessible via the NCBI Gene Expression Omnibus (GEO) database [65] with experiment series accession number GSE98596. To investigate the expression changes causing the reversion of the mesenchymal phenotype seen in the 2APB treated cells, we looked for genes whose expression changes when treated with TGF-β were reversed by the addition of 2APB. First, we calculated the ratio of the expression log2 fold change values (as calculated by DESeq2) of the TGF-β to DMSO treated cells and the TGF-β to TGF-β+2APB treated cells. We reasoned that values of this ratio around 1, with a sign change, would indicate expression values that had changed in one direction upon treatment with TGF-β, and then changed a similar magnitude in the opposite direction when adding the 2APB treatment. We filtered out genes with a mean expression value less than 10 normalized counts to remove noise in the data set. A range of ratio values of –1.5 to –0.5 was chosen for further analysis to catch the bulk of the distribution around –1.0 relating most accurately to the effect we were trying to capture. As above, hierarchical clustering of Pearson correlation values was performed using the aheatmap function in the R package NMF v0.20.6 [62] and network mapping and functional analyses were generated through IPA.

### Protein isolation and Immunoblotting

Proteins were extracted by lysing cell pellets in urea lysis buffer (8 M urea, 1%SDS in Tris-HCl pH 6.5) containing Complete^™^ protease inhibitors (Roche) and phosphatase inhibitors (Sigma- Aldrich), and subsequent heating to 95° C for 5 min. Protein concentration was estimated using the Qubit (Thermo Scientific) protein assay kit, following the manufacturer's instructions. Western blotting was performed as previously described [43, 56], using the following antibodies: anti-SNAI1 (Cell signaling, mouse mAb #3895), anti-phosho-AKT (Ser 473) (Cell signaling, rabbit pAb #9271), anti-total-AKT(Cell signaling, rabbit pAb #9272), anti- Phospho-NF-κB p65 (Ser536) (Cell signaling, rabbit mAb #3033), anti-total-NF-ΚB p65 (Cell Signaling, rabbit mAb #8242), anti- STIM1 (Cell Signaling, rabbit mAb # 5668), anti-STIM2 (Proteintech, rabbit pAb #211921-1-AP), anti-ORAI1 (Proteintech, rabbit pAb #13130-1-AP), anti-ORAI3 (Genetex, rabbit pAb #GTX85677: this antibody worked best with mouse samples) anti-ORAI3 (BosterBio, rabbit pAb A09399; this antibody worked best for human samples) with a dilution of 1:1000 in 5% non-fat dried milk (5% BSA for the antibodies specific to phosphorylated proteins) in 0.1% TBS-T. Anti-GAPDH (Millipore, rabbit pAb # ABS16) was used with a dilution of 1:5,000 in 5% non-fat dried milk in 0.1% TBS-T. Secondary antibodies anti-rabbit IgG, peroxidase-linked species-specific whole antibody (from donkey)-45-001-276(GE Healthcare) and anti-mouse IgG, peroxidase-linked-species-specific whole antibody (from sheep; 45-001-275; GE Healthcare) were diluted 1:10,000 in 5% non-fat dried milk in 0.1% TBST and blots were developed using the Li-COR Odyssey instrument (Li-COR Biosciences) using Luminata Forte Western HRP substrate (WBLUF0500). Western blots presented are representative of at least 3-4 biological replicates, and were quantified using Licor software, and signal normalized to GAPDH, ACTB or total p65/total AKT as denoted for each figure. Statistical analyses were performed with GraphPad Prism software.

### Chromatin Immunoprecipitation (ChIP)

ChIP was performed as previously described [44, 56] with the following exceptions. In brief, cells were crosslinked with 1% formaldehyde for 5 minutes at 37° C, quenched with 2 M glycine and washed with PBS, and then sonicated in the Covaris S220 sonicator (fill level = 8, peak power =120, duty factor = 3, cycles/burst = 200) to generate 300-600bp DNA fragments. Immunoprecipitation was performed using the antibodies indicated, and IgG was used as a control. Precipitated DNAs were detected by PCR using specific primers (see Supplementary Table 1). Quantitation was performed on immunoprecipitated DNA using the CFX384 real-time PCR machine (Bio-Rad) with SYBR-green, and the percent input for each sample was calculated based on a standard curve using 10%, 1%, 0.1% and 0.01% of input DNA.

### Calcium measurements

Cells were incubated with 2 μM fura-2 (Molecular Probes) for 45 min, washed twice with calcium free SES (Standard External Solution, include: 10 mM HEPES, 120 mM NaCl, 5.4 mM KCl, 1 mM MgCl_2_, 10 mM glucose, pH 7.4) buffer. For fluorescence measurements, the fluorescence intensity of Fura-2-loaded control cells was monitored with a CCD camera-based imaging system (Compix) mounted on an Olympus XL70 inverted microscope equipped with an Olympus 40× (1.3 NA) objective. A monochrometer dual wavelength enabled alternative excitation at 340 and 380 nm, whereas the emission fluorescence was monitored at 510 nm with an Orca Imaging camera (Hamamatsu, Japan). The images of multiple cells collected at each excitation wavelength were processed using the C imaging, PCI software (Compix Inc., Cranbery, PA), to provide ratios of Fura-2 fluorescence from excitation at 340 nm to that from excitation at 380 nm (F340/F380). Dispersed cells were placed on glass-bottom poly-D lysine plates and used for the study. Fluorescence traces shown represent [Ca^2+^]_i_ values that are averages from at least 30–40 cells and are a representative of results obtained in at least 3–4 individual experiments.

### Migration assays

Confluent cells in a 6-well plate were serum starved for 4 h prior to treatment, and TGF-β (for 8 h) and/or 2APB (for 24 h) were added to the wells prior to wounding using a sterile 200 ul tip. Three representative fields were marked and imaged immediately at time of (0 h) and a time period after (8 h) wounding as described [66, 67]. Cell migration across the wound was analyzed using ImageJ with MRI Wound healing plugin [68]. The tool measures the area of the wound, i.e. the area that does not contain cells, in each image. A ratio of the area of the wound at the start of wounding and at the end of wound closure is estimated as the percent of cell migration. Data are the average of at least 4 independent experiments, and statistical analyses were performed using GraphPad Prism 7 software.

### Statistical analysis

All statistical analyses for RT-PCR, ChIP and migration assays were performed using GraphPad Prism 7 (GraphPad Software). Data were expressed as mean ± standard error of the mean (S.E.M). The statistical correlation of data between groups was analyzed by a two-tailed Student's *t*-test, where *P <* 0.05 was considered significant.

## SUPPLEMENTARY MATERIALS FIGURES AND TABLES







## Abbreviations

EMT: epithelial to mesenchymal transition
TGF-β: Transforming growth factor beta
2APB: 2-aminoethoxydiphenyl borate
SKF: SKF96365 hydrochloride
DMSO: dimethylsulfoxide
Orai: Orai Calcium Release-Activated Calcium Modulator
STIM: Stromal Interaction Molecule.
