# Supplementary material for: The calcium channel proteins ORAI3 and STIM1 mediate TGF-β induced Snai1 expression
Source: Oncotarget. 2018 Jun 29;9(50):29468–83. doi: 10.18632/oncotarget.25672 (PMC6047677; doi:10.18632/oncotarget.25672)
Supplement: Supplementary file 1 [file oncotarget-09-29468-s001.pdf]

The calcium channel proteins ORAI3 and STIM1 mediate TGF-β induced *Snai1* expression

SUPPLEMENTARY MATERIALS

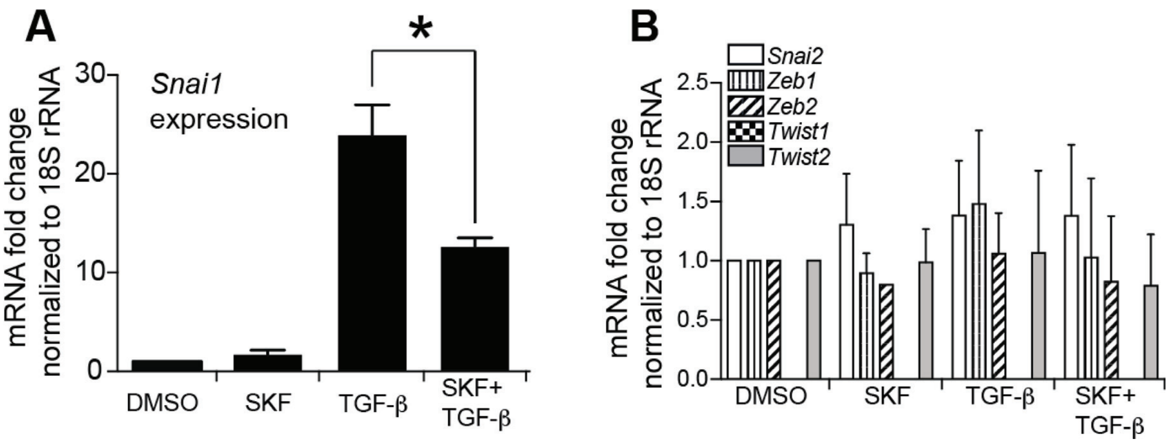

Supplementary Figure 1: The SOCE inhibitor SKF96365 decreases TGF-β induction of *Snai1* gene expression, but not other EMT TFs. NMuMG cells were serum-starved for 4 h, and then treated with DMSO, TGF-β, SKF or TGF-β+SKF for 24 h. RNA was isolated from NMuMG cells and cDNA prepared using reverse transcription. Expression of *Snai1* (**A**) or other EMT genes (**B**) was examined by real-time PCR of the cDNA using primers against each of the genes and normalized to 18S rRNA. Data were derived from at least three independent biological replicates, and are represented as mean ± SEM values. The * indicates *p*-value of ≤ 0.05, ** indicates *p*-value ≤ 0.01 and *** indicates *p*-value ≤ 0.001 as measured by a paired *t*-test.

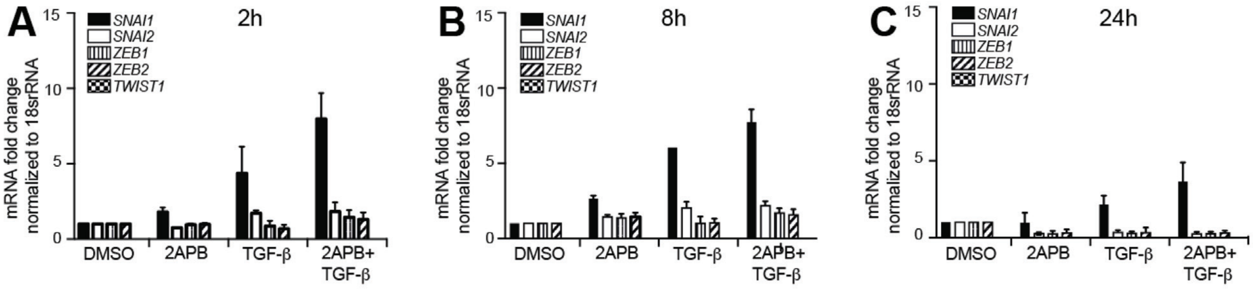

Supplementary Figure 2: Blocking SOCE with 2-APB causes increase in *Snail* transcription following TGF-β treatment in MDA-MB-231 cells. MDA-MB-231 cells were serum-starved for 4 h, and then treated with DMSO or 2APB for 24 h and TGF-β for 2 (**A**), 8 (**B**) and 24 (**C**) hours. RNA was isolated from MDA-MB-231 cells and cDNA prepared using reverse transcription. Expression of EMT genes was examined by real-time PCR of the cDNA using primers against each of the genes and normalized to *18S rRNA*. Data were derived from at least three independent biological replicates, and are represented as mean ± SEM values. The * indicates *p*-value of ≤ 0.05, ** indicates *p*-value ≤ 0.01 and *** indicates *p*-value ≤ 0.001 as measured by a paired *t*-test.

A

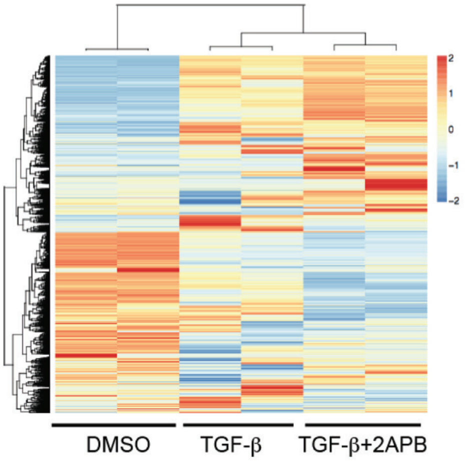

B

Comparison	Upregulated genes	Downregulated genes	Total
TGF-β vs DMSO	3105	2887	5992
TGF-β+2APB vs DMSO	3671	3655	7326
TGF-β+2APB vs TGF-β	1408	1858	3266

C

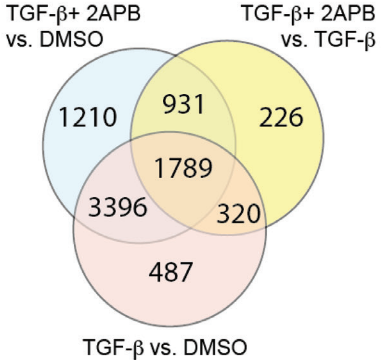

D

IPA Top 5 Molecular and Cellular Functions

TGF-β vs DMSO

Name	p-value	#Molecules
Cellular Movement	2.96E-10 - 1.55E-62	563
Cellular Growth and Proliferation	3.17E-10 - 3.74E-45	829
Cellular Development	4.01E-10 - 1.51E-42	780
Cell-To-Cell Signaling and Interaction	1.11E-10 - 2.06E-34	424
Cell Death and Survival	1.77E-10 - 2.51E-34	678

TGF-β+2APB vs DMSO

Name	p-value	#Molecules
Cellular Movement	9.52E-11 - 1.70E-69	743
Cellular Development	1.05E-10 - 2.92E-52	1059
Cellular Growth and Proliferation	1.05E-10 - 6.83E-49	1116
Cell Death and Survival	1.19E-10 - 9.20E-40	934
Cell-To-Cell Signaling and Interaction	9.20E-11 - 5.14E-37	516

TGF-β+2APB vs TGF-β

Name	p-value	#Molecules
Cellular Development	3.89E-06 - 4.50E-26	364
Cellular Growth and Proliferation	3.89E-06 - 5.73E-26	407
Cellular Movement	3.84E-06 - 1.74E-24	264
Cell Death and Survival	3.84E-06 - 1.44E-22	340
Cell-To-Cell Signaling and Interaction	1.35E-06 - 9.26E-19	184

Supplementary Figure 3: Differential gene expression changes following TGF-β and TGF-β+2-APB. RNA isolated from NMuMG cells treated with DMSO, TGF-β or TGF-β+2APB in triplicate, and two replicates was sequenced using massively parallel sequencing (see text for details). The results were plotted as a heat map to reveal significant differences in gene expression (**A**), and differentially expressed genes (**B**) from each comparison represented as a Venn Diagram (**C**). IPA analysis (**D**) revealed a number of cellular movement, signaling and cell-adhesion pathways were affected.

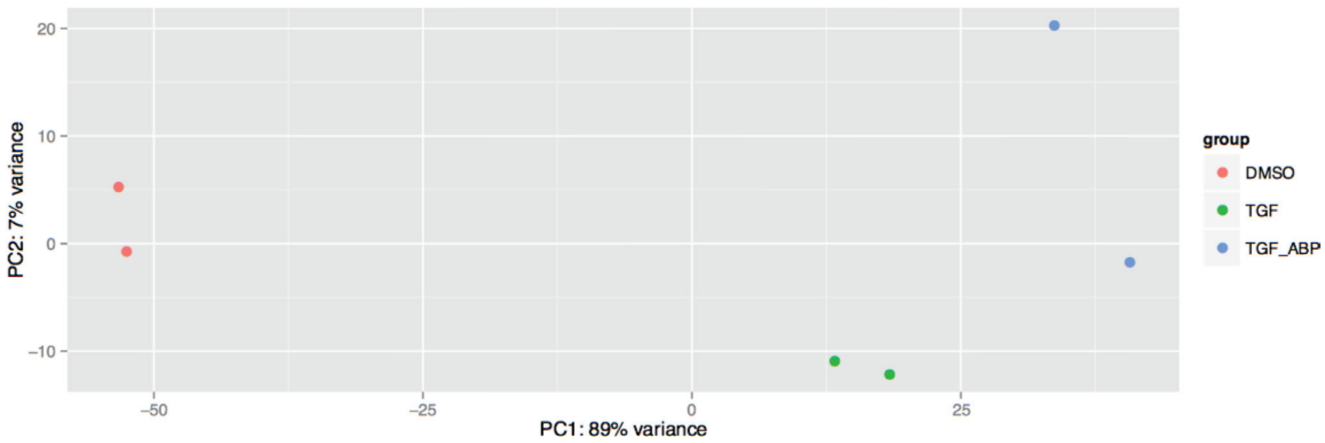

Supplementary Figure 4: Prinicipal component analysis plot of RNA seq data. A Prinicipal component analysis (PCA) plot was created from the RNA-seq data (DMSO, TGF-β and 2APB+TGF-β treatment groups). The plot indicated that the replicates clustered together by treatment groups with no outliers. DMSO treated samples are seen in red, TGF-β treatments in green and TGF-β+2APB treatments in blue.

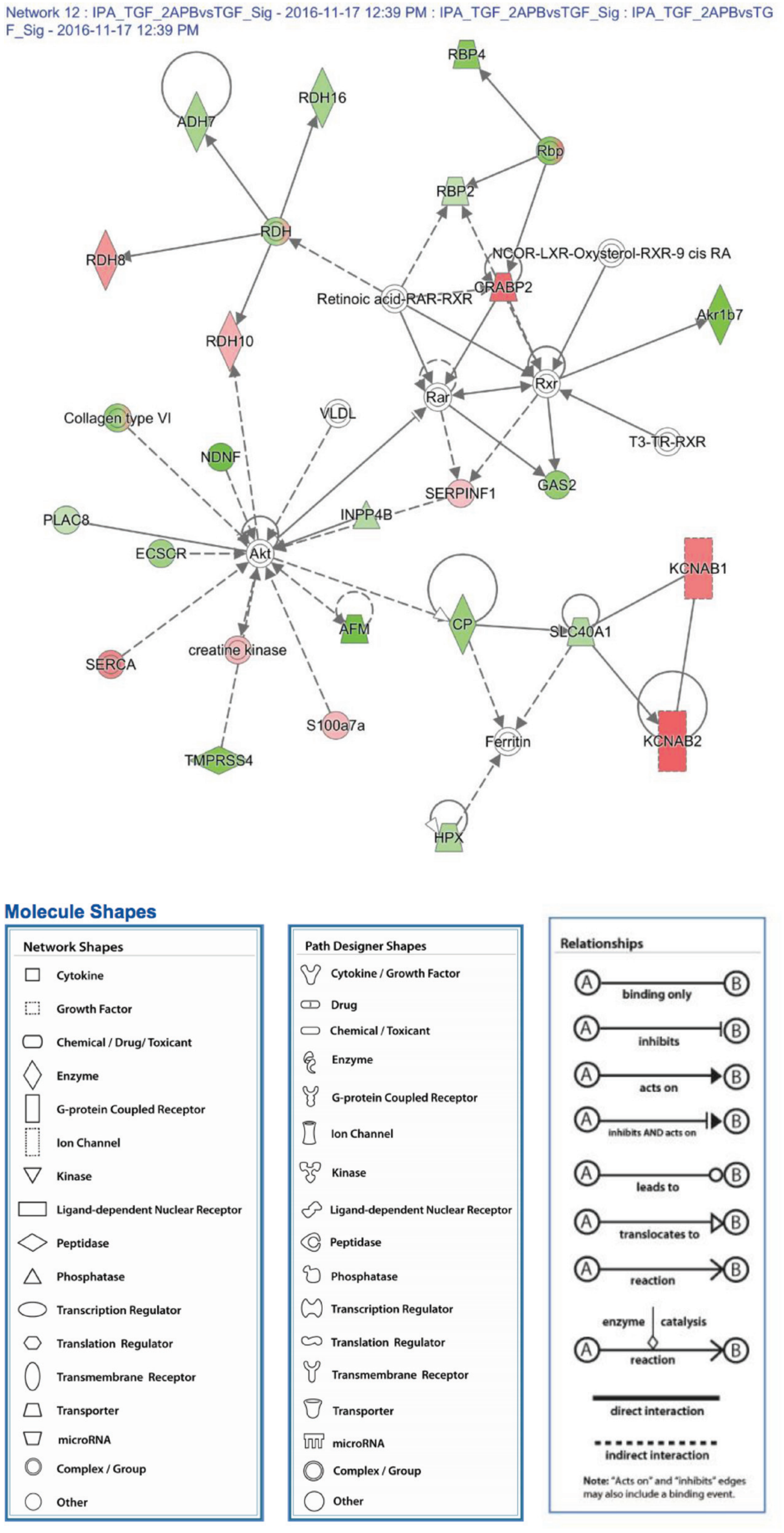

Supplementary Figure 5: AKT network is differentially regulated when comparing TGF-β to 2APB+TGF-β. The RNA-seq expression value changes (Supplementary File 1) were uploaded to the Ingenuity Pathway Analysis website and were analyzed by their proprietary software. The IPA Network Generation Algorithm created networks (Key shown above from the IPA website). One of the top networks thus generated was the AKT pathway (shown here).

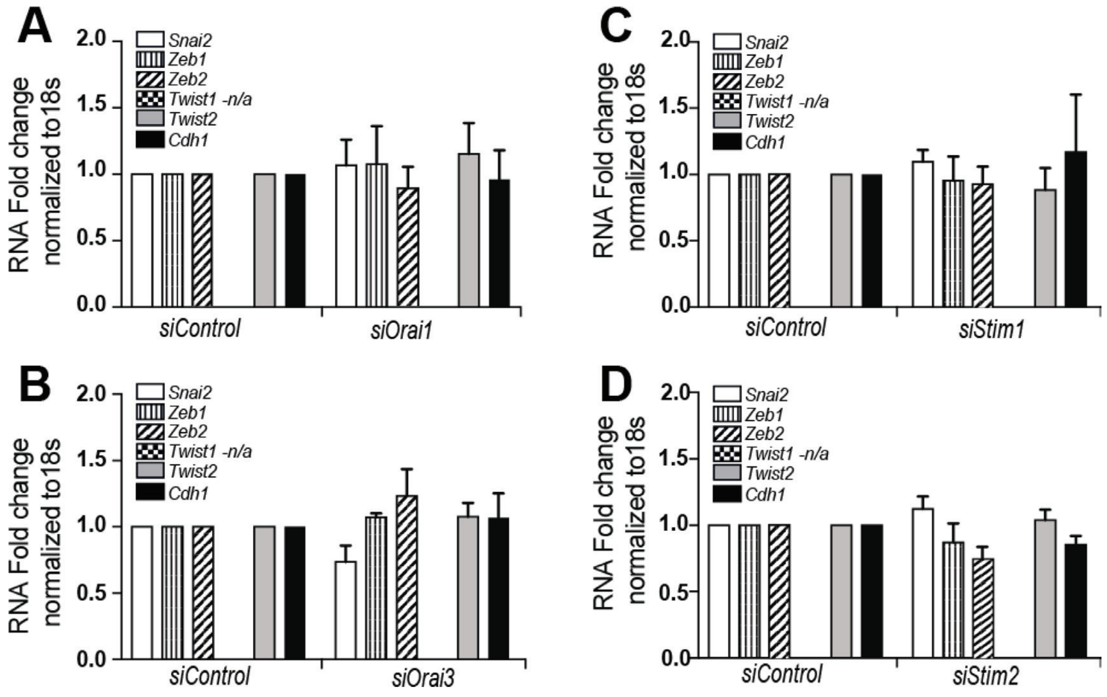

Supplementary Figure 6: EMT transcription factor expression is unaffected by calcium channel knockdowns. The cDNA from the experiment in Figure 4 (NMuMG cells were transfected with *siRNAs* for 96 h) was used to examine transcript levels of EMT genes using RT-PCR. Data were derived from at least three independent biological replicates, and are represented as mean ± SEM values. No Ct values were obtained with *Twist1* primers.

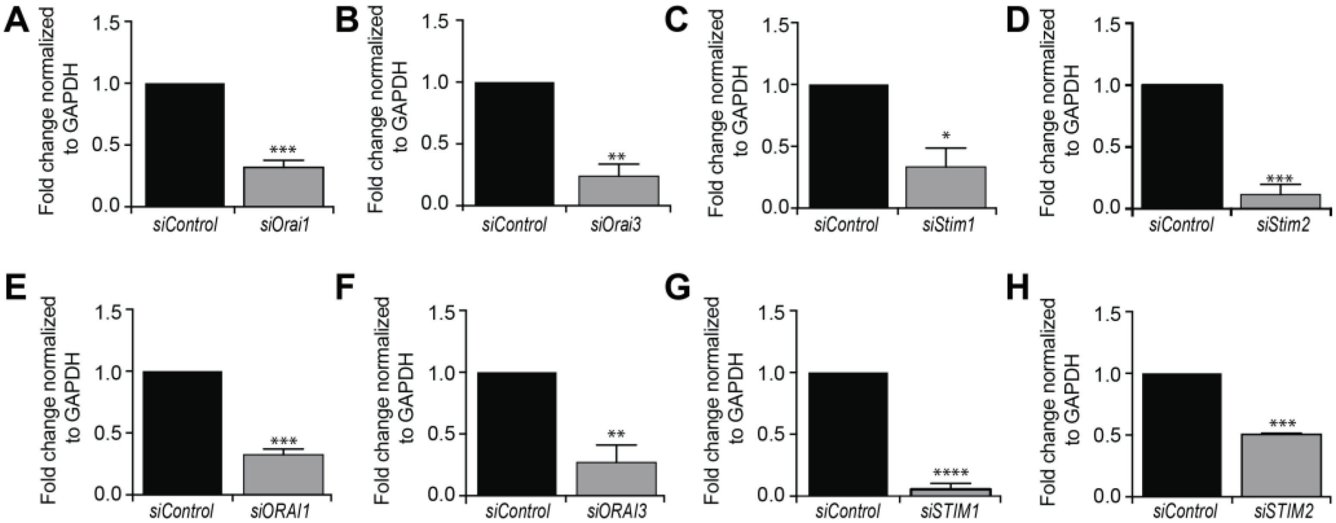

Supplementary Figure 7: Calcium channel knockdown immunoblot quantitation. Three biological replicates of western blots from the experiment in Figure 4 (NMuMG (**A–D**) and MDA-MB-231 (**E–H**) cells transfected with indicated *siRNAs* for 96 h) were quantitated using the LiCoR Fc imaging software. Data represent the average of three independent biological replicates, and are represented as mean ± SEM values.

Supplementary Table 1: List of primers and sequences used in RT-PCR and ChIP experiments

**IDT RT PRIMERS**	
Mm_Twist2_RT_F	cgctacagcaagaaatcgagc
Mm_Twist2_RT_R	gctgagcttgtcagagggg
Mm_Zeb1_RT_F	ccatacgaatgcccgaactg
Mm_Zeb1_RT_R	aacactgagatgtcttgagtcct
Mm_Zeb2_RT_F	agagcttgaccaccgactcaa
Mm_Zeb2_RT_R	ttgcaggactgccttgatctc
Mm_Orai1_RT_F	atgagcctcaacgagcact
Mm_Orai1_RT_R	gtgggtagtcatggtctg
Mm_Orai3_RT_F	gtaccgggagttcgtgca
Mm_Orai3_RT_R	ggtattcatgatcgttctc
Mm_Stim1_RT_F	accgaagcagagttttgccga
Mm_Stim1_RT_F	tcttcccttaggaactcatcact
Mm_Stim2_RT_F	actggagcaggtccgcatg
Mm_Stim2_RT_F	tctgcctcgtccttagcgat
Hs_ORAI1_RT_F	tactccgaggtgatgagcct
Hs_ORAI1_RT_R	acctccaccattgccaccat
Hs_ORAI3_RT_F	ctgccttgctctcgggctt
Hs_ORAI3_RT_R	gacacgtggagaccatgagt
Hs_STIM1_RT_F	gagttggagcaggttcggg
Hs_STIM1_RT_R	ttgcacctccacctcatgtgt
Hs_STIM2_RT_F	ttggacctctaacacgccca
Hs_STIM2_RT_R	tttcaagcctctcctgtaagtc
**Qiagen quantitect primer assays**	
Hs_SNAI1_1_SG	QT00010010
Hs_SNAI2_1_SG	QT00044128
Hs_ZEB1_2_SG	QT01888446
Hs_ZEB2_1_SG	QT00008554
Hs_TWIST1_1_SG	QT00011956
Hs_CDH1_1_SG	QT00080143
Hs_GAPDH_1_SG	QT00079247
Hs_RRN18S_1_SG	QT00199367
Mm_Snai1_1_SG	QT00240940
Mm_Snai2_1_SG	QT00098273
Mm_Twist1_1_SG	QT00097223
Mm_Cdh1_1_SG	QT00121163
Mm_Rn18s_3_SG	QT02448075
Mm_Gapdh_3_SG	QT01658692
**ChIP primers**	
Snai1_ChIP_U1_F	aggatgcccgtaccttagtg
Snai1_ChIP_U1_R	tggccatgcagctcagagat
Snai1_ChIP_U2_F	ggaagaactctggcctttca
Snai1_ChIP_U2_R	agcatccctgagatcaatcc
Snai1_ChIP_P1_F	tgtttattctgtctgtctctctct
Snai1_ChIP_P1_R	agccagaaagtgcgatgatatg
Snai1_ChIP_prom_F	tacctaggtcgctctggccaacat
Snai1_ChIP_prom_R	cgtcctgcagctcgctatagt
Snai1_ChIP_P2_F	ttcaccttccagcagccct
Snai1_ChIP_P2_R	gacagcgaggtcagctcta
Snai1_ChIP_P3_F	gacctgtggaaaggccttct
Snai1_ChIP_P3_R	aatggagaacaaccaacatctact

Supplementary File 1: Excel sheet with list of differentially expressed genes from the RNA-seq experiment. Each tab represents a different comparison. See Supplementary_File_1

Supplementary File 2: IPA analysis summary of significant differentially expressed genes. See Supplementary_File_2
