# Supplementary material for: The calcium channel proteins ORAI3 and STIM1 mediate TGF-β induced Snai1 expression
Source: Oncotarget. 2018 Jun 29;9(50):29468–83. doi: 10.18632/oncotarget.25672 (PMC6047677; doi:10.18632/oncotarget.25672)
Supplement: Supplementary file 3 [file oncotarget-09-29468-s003.pdf]

INGENUITY
PATHWAY ANALYSIS

Analysis Name: IPA_TGFvsDMSO_Sig - 2016-11-17 12:38 PM
Analysis Creation Date: 2016-11-17
Build version: 400896M
Content version: 28820210 (Release Date: 2016-09-24)

Analysis Settings

Reference set: Ingenuity Knowledge Base (Genes Only)
Relationship to include: Direct and Indirect
Includes Endogenous Chemicals
Optional Analyses: My Pathways My List

Filter Summary:
Consider only relationships where
confidence = Experimentally Observed

(c) 2000-2016 QIAGEN. All rights reserved.

Top Canonical Pathways

Name	p-value	Overlap
LPS/IL-1 Mediated Inhibition of RXR Function	1.63E-18	29.0 % 64/221
Hepatic Fibrosis / Hepatic Stellate Cell Activation	1.42E-15	29.0 % 53/183
LXR/RXR Activation	9.07E-13	31.4 % 38/121
Xenobiotic Metabolism Signaling	1.06E-12	22.4 % 64/286
FXR/RXR Activation	3.68E-12	30.2 % 38/126

Top Upstream Regulators

Upstream Regulator	p-value of overlap	Predicted Activation
TGFB1	6.47E-70	Activated
TNF	2.03E-62	
dexamethasone	7.17E-54	Inhibited
beta-estradiol	2.85E-51	
lipopolysaccharide	5.35E-45	

Top Diseases and Bio Functions

Diseases and Disorders

Name	p-value	#Molecules
Cancer	4.01E-10 - 2.81E-43	1682
Organismal Injury and Abnormalities	4.01E-10 - 2.81E-43	1727
Gastrointestinal Disease	3.49E-10 - 9.89E-42	1543
Reproductive System Disease	3.08E-10 - 4.86E-30	985
Dermatological Diseases and Conditions	3.64E-22 - 3.45E-29	825

Molecular and Cellular Functions

(c) 2000-2016 QIAGEN. All rights reserved.

INGENUITY PATHWAY ANALYSIS

Name	p-value	#Molecules
Cellular Movement	2.96E-10 - 1.55E-62	563
Cellular Growth and Proliferation	3.17E-10 - 3.74E-45	829
Cellular Development	4.01E-10 - 1.51E-42	780
Cell-To-Cell Signaling and Interaction	1.11E-10 - 2.06E-34	424
Cell Death and Survival	1.77E-10 - 2.51E-34	678

Physiological System Development and Function

Name	p-value	#Molecules
Organismal Development	3.17E-10 - 1.34E-40	793
Cardiovascular System Development and Function	3.79E-10 - 2.44E-40	406
Tissue Morphology	3.90E-10 - 1.11E-37	584
Organismal Survival	1.64E-24 - 8.49E-33	540
Tissue Development	3.17E-10 - 1.14E-27	757

Top Tox Functions

Assays: Clinical Chemistry and Hematology

Name	p-value	#Molecules
Increased Levels of Alkaline Phosphatase	2.38E-01 - 1.09E-04	18
Increased Levels of Hematocrit	1.55E-04 - 1.55E-04	20
Decreased Levels of Albumin	4.19E-01 - 2.43E-03	10
Increased Levels of AST	4.19E-01 - 6.96E-03	6
Increased Levels of Red Blood Cells	5.30E-02 - 4.00E-02	14

Cardiotoxicity

(c) 2000-2016 QIAGEN. All rights reserved.

Name	p-value	#Molecules
Cardiac Dysfunction	4.70E-01 - 2.68E-14	54
Cardiac Hypertrophy	2.38E-01 - 3.11E-12	82
Cardiac Congestive Cardiac Failure	2.29E-08 - 2.29E-08	30
Heart Failure	1.66E-01 - 2.29E-08	54
Cardiac Fibrosis	4.70E-01 - 5.53E-07	42

Hepatotoxicity

Name	p-value	#Molecules
Liver Hyperplasia/Hyperproliferation	5.58E-01 - 8.60E-18	737
Liver Proliferation	2.38E-01 - 2.41E-10	52
Liver Cirrhosis	1.66E-01 - 3.71E-10	57
Hepatocellular Carcinoma	5.58E-01 - 3.77E-09	142
Liver Cholestasis	4.70E-01 - 4.55E-09	30

Nephrotoxicity

Name	p-value	#Molecules
Kidney Failure	5.54E-01 - 2.70E-12	60
Renal Damage	1.66E-01 - 2.81E-09	61
Glomerular Injury	3.64E-01 - 3.75E-08	71
Renal Proliferation	4.70E-01 - 7.87E-08	59
Renal Necrosis/Cell Death	5.16E-01 - 1.15E-07	88

Top Regulator Effect Networks

ID	Regulators	Diseases & Functions	Consistency Score
1	AIF1,EPHB6,FASN,POSTN,SCUBE3	binding of protein binding site (+10 more)	27.106

(c) 2000-2016 QIAGEN. All rights reserved.

INGENUITY PATHWAY ANALYSIS

2	AREG,EREG,FGFR1,mir-15,PCDH11Y,PRKCE,RET,TGFA	central nervous system tumor (+8 more)	22.791
3	AREG,FOXC2,HMGA2,ILK,MBD2,mir-15,PDCD4,POSTN,RET (+1 more)	cell spreading,central nervous system tumor (+8 more)	19.147
4	AREG,mir-1,RET,TGFA	cell movement of carcinoma cell lines (+5 more)	18.499
5	AREG,EREG,FASN,FGFR1,FOXC2,mir-15,PDCD4,PRKCE,RET (+2 more)	central nervous system tumor (+5 more)	18.357

Top Networks

ID	Associated Network Functions	Score
1	Hereditary Disorder, Neurological Disease, Organismal Injury and Abnormalities	38
2	Connective Tissue Disorders, Developmental Disorder, Hereditary Disorder	36
3	Cellular Development, Hematological System Development and Function, Hematopoiesis	36
4	Amino Acid Metabolism, Small Molecule Biochemistry, Lipid Metabolism	36
5	Cellular Development, Cellular Growth and Proliferation, Organ Development	34

Top Tox Lists

Name	p-value	Overlap	
LPS/IL-1 Mediated Inhibition of RXR Function	3.47E-20	28.5 %	72/253
Xenobiotic Metabolism Signaling	1.97E-15	22.4 %	79/352
LXR/RXR Activation	1.60E-12	30.9 %	38/123
Cardiac Hypertrophy	1.71E-12	19.2 %	85/442
FXR/RXR Activation	3.68E-12	30.2 %	38/126

Top Analysis-Ready Molecules

Exp Log Ratio up-regulated

Molecules	Exp. Value	Exp. Chart
**Lce1g**	↑ 8.189	

(c) 2000-2016 QIAGEN. All rights reserved.

**IL11**	↑ 7.771	
**CTSW**	↑ 7.584	
**IGLON5**	↑ 7.384	
**LGR6**	↑ 7.369	
**PKP1**	↑ 7.093	
**PCOLCE2**	↑ 7.001	
**TMEM119**	↑ 6.921	
**COL7A1**	↑ 6.844	
**NTSR1**	↑ 6.832	

Exp Log Ratio down-regulated

Molecules	Exp. Value	Exp. Chart
**AGT**	↓ -7.940	
**GSTA5***	↓ -7.592	
**ALB**	↓ -7.547	
**APOA1**	↓ -7.376	
**Pzp**	↓ -7.201	
**AFM**	↓ -7.195	
**CYP4A22***	↓ -7.190	
**Cyp2c70**	↓ -7.152	
**TTR**	↓ -6.738	
**Sult1d1**	↓ -6.725	

(c) 2000-2016 QIAGEN. All rights reserved.

INGENUITY PATHWAY ANALYSIS

Analysis Name: IPA_TGF_2APBvsDMSO_Sig - 2016-11-17 01:12 PM
Analysis Creation Date: 2016-11-17
Build version: 400896M
Content version: 28820210 (Release Date: 2016-09-24)

Analysis Settings

Reference set: Ingenuity Knowledge Base (Genes Only)
Relationship to include: Direct and Indirect
Includes Endogenous Chemicals
Optional Analyses: My Pathways My List

Filter Summary:
Consider only relationships where
confidence = Experimentally Observed

(c) 2000-2016 QIAGEN. All rights reserved.

Top Canonical Pathways

Name	p-value	Overlap	
LPS/IL-1 Mediated Inhibition of RXR Function	4.76E-17	33.9 %	75/221
Xenobiotic Metabolism Signaling	2.64E-13	28.3 %	81/286
LXR/RXR Activation	5.22E-13	38.0 %	46/121
Hepatic Fibrosis / Hepatic Stellate Cell Activation	1.25E-12	32.2 %	59/183
Acute Phase Response Signaling	5.89E-10	30.2 %	51/169

Top Upstream Regulators

Upstream Regulator	p-value of overlap	Predicted Activation
TGFB1	1.63E-73	Activated
TNF	4.71E-70	
dexamethasone	1.12E-60	Inhibited
beta-estradiol	7.67E-57	
progesterone	1.42E-46	

Top Diseases and Bio Functions

Diseases and Disorders

Name	p-value	#Molecules
Cancer	1.31E-10 - 1.21E-59	2388
Organismal Injury and Abnormalities	1.40E-10 - 1.21E-59	2452
Gastrointestinal Disease	1.31E-10 - 3.19E-50	2183
Reproductive System Disease	9.79E-11 - 5.59E-30	1315
Inflammatory Response	2.08E-11 - 1.02E-28	727

Molecular and Cellular Functions

(c) 2000-2016 QIAGEN. All rights reserved.

INGENUITY PATHWAY ANALYSIS

Name	p-value	#Molecules
Cellular Movement	9.52E-11 - 1.70E-69	743
Cellular Development	1.05E-10 - 2.92E-52	1059
Cellular Growth and Proliferation	1.05E-10 - 6.83E-49	1116
Cell Death and Survival	1.19E-10 - 9.20E-40	934
Cell-To-Cell Signaling and Interaction	9.20E-11 - 5.14E-37	516

Physiological System Development and Function

Name	p-value	#Molecules
Tissue Morphology	1.06E-10 - 4.29E-45	769
Organismal Development	1.07E-10 - 4.90E-44	1018
Cardiovascular System Development and Function	1.12E-10 - 5.29E-44	514
Immune Cell Trafficking	9.20E-11 - 8.97E-34	391
Hematological System Development and Function	9.20E-11 - 2.73E-33	642

Top Tox Functions

Assays: Clinical Chemistry and Hematology

Name	p-value	#Molecules
Increased Levels of Alkaline Phosphatase	3.27E-01 - 3.31E-04	21
Increased Levels of Hematocrit	1.04E-03 - 1.04E-03	23
Decreased Levels of Albumin	5.47E-01 - 6.83E-03	9
Increased Levels of AST	5.47E-01 - 2.96E-02	6
Increased Levels of ALT	4.83E-01 - 3.71E-02	9

Cardiotoxicity

(c) 2000-2016 QIAGEN. All rights reserved.

INGENUITY PATHWAY ANALYSIS

Name	p-value	#Molecules
Cardiac Hypertrophy	3.27E-01 - 1.14E-12	105
Cardiac Dysfunction	3.08E-01 - 4.72E-11	62
Heart Failure	4.10E-01 - 1.14E-08	74
Cardiac Infarction	2.59E-01 - 2.57E-08	69
Cardiac Fibrosis	2.11E-01 - 1.90E-07	54

Hepatotoxicity

Name	p-value	#Molecules
Liver Hyperplasia/Hyperproliferation	6.05E-01 - 9.83E-24	1040
Liver Proliferation	3.27E-01 - 4.50E-16	77
Hepatocellular Carcinoma	6.05E-01 - 9.25E-13	202
Liver Steatosis	3.27E-01 - 5.98E-11	90
Liver Fibrosis	6.52E-01 - 8.09E-11	70

Nephrotoxicity

Name	p-value	#Molecules
Kidney Failure	1.00E00 - 2.79E-10	70
Renal Damage	2.32E-01 - 1.06E-09	73
Renal Proliferation	3.27E-01 - 2.75E-09	77
Glomerular Injury	4.83E-01 - 3.65E-09	86
Renal Necrosis/Cell Death	6.52E-01 - 9.81E-08	114

Top Regulator Effect Networks

ID	Regulators	Diseases & Functions	Consistency Score
1	Collagen Alpha1,HDAC7,PDCD4	adhesion of blood cells,development of vasculature (+5 more)	17.907

(c) 2000-2016 QIAGEN. All rights reserved.

2	CD3E,Collagen Alpha1,Collagen type I,DEF6,ILK (+5 more)	development of neurons (+5 more)	16.045
3	CD3E,Collagen Alpha1,Collagen type I,DEF6,PTAFR (+1 more)	development of connective tissue (+6 more)	14.892
4	EPHB6,GSC	differentiation of adipocytes (+6 more)	13.88
5	AQP11,PDGFC,PPBP	binding of protein binding site (+6 more)	10.681

Top Networks

ID	Associated Network Functions	Score
1	Auditory Disease, Hereditary Disorder, Neurological Disease	33
2	Connective Tissue Development and Function, Connective Tissue Disorders, Nervous System Development and Function	33
3	Cellular Development, Cellular Growth and Proliferation, Organ Development	31
4	Developmental Disorder, Hereditary Disorder, Metabolic Disease	31
5	Cellular Development, Cellular Growth and Proliferation, Embryonic Development	31

Top Tox Lists

Name	p-value	Overlap	
LPS/IL-1 Mediated Inhibition of RXR Function	1.06E-17	32.8 %	83/253
Liver Proliferation	3.32E-17	33.6 %	77/229
Xenobiotic Metabolism Signaling	7.82E-15	27.6 %	97/352
LXR/RXR Activation	1.04E-12	37.4 %	46/123
Cardiac Hypertrophy	1.35E-12	24.4 %	108/442

Top Analysis-Ready Molecules

Exp Log Ratio up-regulated

Molecules	Exp. Value	Exp. Chart
**Lce1g**	↑ 9.610	

(c) 2000-2016 QIAGEN. All rights reserved.

INGENUITY PATHWAY ANALYSIS

**IGLON5**	↑ 8.979	
**IL11**	↑ 8.602	
**NTSR1**	↑ 8.183	
**ANGPTL2**	↑ 8.142	
**NAT8L**	↑ 8.095	
**PKP1**	↑ 7.730	
**LGR6**	↑ 7.667	
**CTSW**	↑ 7.599	
**CLDN4**	↑ 7.576	

Exp Log Ratio down-regulated

Molecules	Exp. Value	Exp. Chart
**AFM**	↓ -10.453	
**CYP4A22***	↓ -10.231	
**Sult1d1**	↓ -8.849	
**AGT**	↓ -8.689	
**APOA1**	↓ -8.544	
**ALB**	↓ -8.398	
**FGA**	↓ -8.329	
**Pcp4l1**	↓ -7.994	
**GSTA5***	↓ -7.943	
**Cyp2c70**	↓ -7.787	

(c) 2000-2016 QIAGEN. All rights reserved.

INGENUITY PATHWAY ANALYSIS

INGENUITY
PATHWAY ANALYSIS

Analysis Name: IPA_TGF_2APBvsTGF_Sig - 2016-11-17 12:39 PM
Analysis Creation Date: 2016-11-17
Build version: 400896M
Content version: 28820210 (Release Date: 2016-09-24)

Analysis Settings

Reference set: Ingenuity Knowledge Base (Genes + Endogenous Chemicals)
Relationship to include: Direct and Indirect
Includes Endogenous Chemicals
Optional Analyses: My Pathways My List

Filter Summary:
Consider only relationships where
confidence = Experimentally Observed

(c) 2000-2016 QIAGEN. All rights reserved.

Top Canonical Pathways

Name	p-value	Overlap
LPS/IL-1 Mediated Inhibition of RXR Function	6.11E-10	13.7 % 31/226
Acute Phase Response Signaling	7.50E-09	14.6 % 25/171
LXR/RXR Activation	1.50E-08	16.4 % 21/128
Xenobiotic Metabolism Signaling	6.41E-08	11.1 % 32/289
Nicotine Degradation III	2.47E-07	22.0 % 13/59

Top Upstream Regulators

Upstream Regulator	p-value of overlap	Predicted Activation
lipopolysaccharide	4.63E-32	
dextran sulfate	3.18E-30	
TGFB1	5.50E-30	Activated
IL6	5.98E-27	Inhibited
beta-estradiol	2.06E-26	

Top Diseases and Bio Functions

Diseases and Disorders

Name	p-value	#Molecules
Cancer	3.97E-06 - 8.05E-34	791
Organismal Injury and Abnormalities	4.05E-06 - 8.05E-34	811
Gastrointestinal Disease	4.05E-06 - 3.60E-30	731
Reproductive System Disease	2.00E-06 - 2.54E-23	461
Dermatological Diseases and Conditions	2.37E-06 - 3.71E-23	427

Molecular and Cellular Functions

(c) 2000-2016 QIAGEN. All rights reserved.

Name	p-value	#Molecules
Cellular Development	3.89E-06 - 4.50E-26	364
Cellular Growth and Proliferation	3.89E-06 - 5.73E-26	407
Cellular Movement	3.84E-06 - 1.74E-24	264
Cell Death and Survival	3.84E-06 - 1.44E-22	340
Cell-To-Cell Signaling and Interaction	1.35E-06 - 9.26E-19	184

Physiological System Development and Function

Name	p-value	#Molecules
Tissue Morphology	2.64E-06 - 6.20E-28	273
Hematological System Development and Function	3.89E-06 - 2.33E-24	239
Organismal Development	3.09E-06 - 6.90E-20	343
Lymphoid Tissue Structure and Development	3.89E-06 - 3.70E-19	170
Cardiovascular System Development and Function	2.84E-06 - 5.18E-17	162

Top Tox Functions

Assays: Clinical Chemistry and Hematology

Name	p-value	#Molecules
Increased Levels of Albumin	4.33E-02 - 4.33E-02	2
Decreased Levels of Hematocrit	5.28E-02 - 5.28E-02	2
Increased Levels of ALT	3.47E-01 - 7.41E-02	4
Increased Levels of Bilirubin	1.09E-01 - 1.09E-01	2
Increased Levels of Potassium	1.48E-01 - 1.48E-01	2

Cardiotoxicity

(c) 2000-2016 QIAGEN. All rights reserved.

INGENUITY PATHWAY ANALYSIS

Name	p-value	#Molecules
Cardiac Infarction	2.94E-01 - 1.02E-06	30
Cardiac Hypertrophy	3.95E-01 - 1.18E-05	35
Pulmonary Hypertension	8.71E-03 - 4.07E-05	15
Cardiac Stenosis	8.50E-02 - 2.38E-04	9
Cardiac Output	9.68E-02 - 3.51E-04	8

Hepatotoxicity

Name	p-value	#Molecules
Liver Hyperplasia/Hyperproliferation	5.21E-01 - 3.76E-16	359
Hepatocellular Carcinoma	3.71E-01 - 1.32E-09	80
Liver Steatosis	2.87E-01 - 1.80E-05	33
Liver Fibrosis	3.68E-01 - 3.59E-05	23
Liver Inflammation/Hepatitis	1.00E00 - 3.83E-05	33

Nephrotoxicity

Name	p-value	#Molecules
Kidney Failure	5.56E-01 - 6.97E-08	33
Renal Damage	5.73E-01 - 2.43E-06	28
Renal Tubule Injury	1.61E-01 - 2.43E-06	20
Renal Necrosis/Cell Death	6.34E-01 - 5.59E-05	46
Renal Inflammation	1.00E00 - 4.77E-04	30

Top Regulator Effect Networks

ID	Regulators	Diseases & Functions	Consistency Score
1	ACTL6A,AR,CDKN1A,CDKN2A,CHUK,CSF2,E2F3,ESR1,FOXM1 (+13 more)	cytokinesis of tumor cell lines (+2 more)	14.606

(c) 2000-2016 QIAGEN. All rights reserved.

2	ACTL6A,ATF6,EP400,LHX1,RABL6,Retinoic acid-RAR-RXR (+2 more)	cell proliferation of tumor cell lines (+3 more)	7.031
3	LCN2,MAP3K8,MYBL2,PHLPP1,SOCS6	cell viability of tumor cell lines,obesity	4.714
4	MST1,SMOC2,Stat3-Stat3	apoptosis of tumor cell lines,obesity (+1 more)	3.357
5	DCN,FOXO1,MAP3K8,PTGER2,TGFB2	cerebrovascular dysfunction,obesity	3.212

Top Networks

ID	Associated Network Functions	Score
1	Cell Cycle, Cellular Assembly and Organization, DNA Replication, Recombination, and Repair	47
2	Cell-To-Cell Signaling and Interaction, Hematological System Development and Function, Tissue Morphology	42
3	Cell Cycle, Cellular Movement, DNA Replication, Recombination, and Repair	40
4	Endocrine System Development and Function, Small Molecule Biochemistry, Amino Acid Metabolism	37
5	Post-Translational Modification, Connective Tissue Disorders, Dermatological Diseases and Conditions	34

Top Tox Lists

Name	p-value	Overlap	
LPS/IL-1 Mediated Inhibition of RXR Function	6.48E-10	13.0 %	33/253
LXR/RXR Activation	7.21E-09	17.1 %	21/123
Xenobiotic Metabolism Signaling	7.53E-08	10.2 %	36/352
Positive Acute Phase Response Proteins	8.81E-08	33.3 %	10/30
Renal Necrosis/Cell Death	1.35E-07	8.8 %	46/525

Top Analysis-Ready Molecules

Exp Log Ratio up-regulated

Molecules	Exp. Value	Exp. Chart
Mroh4	↑ 4.246	
**BIRC7**	↑ 4.093	
**CHRNA4**	↑ 3.800	

(c) 2000-2016 QIAGEN. All rights reserved.

**KCNN3**	↑ 3.778
**PRKG1**	↑ 3.574
**FIBCD1**	↑ 3.363
**DLX3**	↑ 3.218
**LTF**	↑ 3.161
**OSM**	↑ 3.155
**Scgb1b27 (includes others)**	↑ 3.139

Exp Log Ratio down-regulated

Molecules	Exp. Value	Exp. Chart
**FGA**	↓ -4.174	
**STC1**	↓ -3.888	
**PCDH10**	↓ -3.864	
**GZMH**	↓ -3.641	
**Mcpt8**	↓ -3.595	
**mir-1247**	↓ -3.563	
**ENPEP**	↓ -3.554	
**NDNF**	↓ -3.330	
**CFH**	↓ -3.265	
**AFM**	↓ -3.258	

(c) 2000-2016 QIAGEN. All rights reserved.

Analysis Name: interval_1.5_Up_then_down_170518 - 2017-05-18 05:16 PM
Analysis Creation Date: 2017-05-18
Build version: 441680M
Content version: 33559992 (Release Date: 2017-03-28)

Analysis Settings

Reference set: Ingenuity Knowledge Base (Genes Only)
Relationship to include: Direct and Indirect
Includes Endogenous Chemicals
Optional Analyses: My Pathways My List

Filter Summary:
Consider only relationships where
confidence = Experimentally Observed

(c) 2000-2017 QIAGEN. All rights reserved.

Summary of Analysis - interval_1.5_Up_then_down_170518 - 2017-05-18 05:16 PM

Top Canonical Pathways

Name	p-value	Overlap	
Role of BRCA1 in DNA Damage Response	4.65E-06	15.4 %	12/78
Pancreatic Adenocarcinoma Signaling	1.72E-05	11.9 %	14/118
DNA Double-Strand Break Repair by Homologous Recombination	4.42E-05	35.7 %	5/14
Molecular Mechanisms of Cancer	1.13E-04	7.0 %	26/374
Dolichyl-diphosphooligosaccharide Biosynthesis	2.51E-04	36.4 %	4/11

Top Upstream Regulators

Upstream Regulator	p-value of overlap	Predicted Activation
E2F4	7.65E-12	
E2f	2.34E-09	
RB1	2.88E-08	
EP400	3.12E-08	
TBX2	1.06E-07	

Top Diseases and Bio Functions

Diseases and Disorders

Name	p-value	#Molecules
Cancer	1.42E-02 - 1.63E-07	586
Organismal Injury and Abnormalities	1.42E-02 - 1.63E-07	591
Gastrointestinal Disease	1.40E-02 - 5.71E-07	485
Developmental Disorder	1.33E-02 - 6.41E-06	131
Neurological Disease	1.32E-02 - 1.04E-05	34

Molecular and Cellular Functions

(c) 2000-2017 QIAGEN. All rights reserved.

Name	p-value	#Molecules
DNA Replication, Recombination, and Repair	1.22E-02 - 1.21E-12	108
Cell Cycle	1.43E-02 - 1.69E-07	155
Cellular Development	1.32E-02 - 2.98E-06	135
Cell Morphology	1.32E-02 - 6.33E-06	137
Cellular Assembly and Organization	1.32E-02 - 6.33E-06	151

Physiological System Development and Function

Name	p-value	#Molecules
Organismal Survival	7.36E-03 - 7.74E-08	162
Embryonic Development	1.37E-02 - 7.35E-07	112
Organismal Development	1.42E-02 - 7.35E-07	155
Tissue Morphology	1.32E-02 - 7.35E-07	80
Connective Tissue Development and Function	1.22E-02 - 2.98E-06	60

Top Tox Functions

Assays: Clinical Chemistry and Hematology

Name	p-value	#Molecules
Increased Levels of Potassium	6.09E-02 - 6.09E-02	1
Increased Levels of Alkaline Phosphatase	1.83E-01 - 1.02E-01	5
Increased Levels of Blood Urea Nitrogen	1.05E-01 - 1.05E-01	2
Decreased Levels of Albumin	1.45E-01 - 1.45E-01	1
Increased Levels of LDH	2.92E-01 - 2.92E-01	1

Cardiotoxicity

(c) 2000-2017 QIAGEN. All rights reserved.

INGENUITY PATHWAY ANALYSIS

Name	p-value	#Molecules
Cardiac Hypoplasia	3.35E-01 - 6.85E-03	7
Cardiac Damage	3.14E-01 - 8.32E-03	6
Cardiac Degeneration	8.97E-03 - 8.97E-03	2
Cardiac Hemorrhaging	8.99E-02 - 1.81E-02	2
Bradycardia	3.86E-01 - 3.09E-02	2

Hepatotoxicity

Name	p-value	#Molecules
Liver Inflammation/Hepatitis	1.00E00 - 2.81E-03	12
Liver Hyperplasia/Hyperproliferation	1.00E00 - 1.03E-02	225
Hepatocellular Carcinoma	5.98E-01 - 1.22E-02	34
Liver Proliferation	2.33E-01 - 1.70E-02	11
Liver Fibrosis	3.80E-01 - 2.36E-02	9

Nephrotoxicity

Name	p-value	#Molecules
Renal Necrosis/Cell Death	5.15E-01 - 4.90E-03	28
Glomerular Injury	5.15E-01 - 1.81E-02	10
Kidney Failure	5.00E-01 - 3.09E-02	7
Nephrosis	4.67E-01 - 3.09E-02	5
Renal Dilation	3.09E-02 - 3.09E-02	1

Top Networks

ID	Associated Network Functions	Score
1	RNA Post-Transcriptional Modification, Nucleic Acid Metabolism, Small Molecule Biochemistry	51
2	DNA Replication, Recombination, and Repair, Cell Cycle, RNA Post-Transcriptional Modification	48

(c) 2000-2017 QIAGEN. All rights reserved.

3	Cellular Assembly and Organization, DNA Replication, Recombination, and Repair, Hereditary Disorder	44
4	Cell Cycle, Cellular Assembly and Organization, DNA Replication, Recombination, and Repair	43
5	DNA Replication, Recombination, and Repair, Cell Cycle, Cellular Compromise	40

Top Tox Lists

Name	p-value	Overlap	
Renal Necrosis/Cell Death	2.49E-03	5.4 %	29/533
Cell Cycle: G1/S Checkpoint Regulation	4.20E-03	10.6 %	7/66
TGF- Signaling	2.15E-02	7.8 %	7/90
Increases Heart Failure	3.29E-02	13.0 %	3/23
Genes Upregulated in Response to Proteinuria-induced Oxidative Stress in Renal Proximal Tubule Cells (Human)	3.64E-02	20.0 %	2/10

(c) 2000-2017 QIAGEN. All rights reserved.

Analysis Name: interval_1.5_down_then_up_170518 - 2017-05-18 05:16 PM
Analysis Creation Date: 2017-05-18
Build version: 441680M
Content version: 33559992 (Release Date: 2017-03-28)

Analysis Settings

Reference set: Ingenuity Knowledge Base (Genes Only)
Relationship to include: Direct and Indirect
Includes Endogenous Chemicals
Optional Analyses: My Pathways My List

Filter Summary:
Consider only relationships where
confidence = Experimentally Observed

(c) 2000-2017 QIAGEN. All rights reserved.

Top Canonical Pathways

Name	p-value	Overlap	
Protein Ubiquitination Pathway	1.27E-04	8.6 %	22/255
Caveolar-mediated Endocytosis Signaling	9.11E-04	12.7 %	9/71
Adenine and Adenosine Salvage I	1.27E-03	100.0 %	2/2
Regulation of eIF4 and p70S6K Signaling	1.50E-03	8.9 %	14/157
Oxidative Phosphorylation	1.76E-03	10.1 %	11/109

Top Upstream Regulators

Upstream Regulator	p-value of overlap	Predicted Activation
HNF4A	6.75E-07	
ST1926	7.43E-06	
RICTOR	1.39E-05	
IL5	3.96E-05	
CD38	5.72E-05	

Top Diseases and Bio Functions

Diseases and Disorders

Name	p-value	#Molecules
Cancer	1.73E-02 - 2.38E-12	685
Organismal Injury and Abnormalities	1.73E-02 - 2.38E-12	697
Gastrointestinal Disease	1.73E-02 - 5.33E-08	568
Reproductive System Disease	1.67E-02 - 5.42E-07	308
Developmental Disorder	1.73E-02 - 2.20E-06	91

Molecular and Cellular Functions

(c) 2000-2017 QIAGEN. All rights reserved.

INGENUITY PATHWAY ANALYSIS

Name	p-value	#Molecules
Cell Morphology	1.73E-02 - 1.23E-05	181
Gene Expression	1.73E-02 - 1.70E-05	157
Cellular Movement	1.47E-02 - 2.83E-05	35
Cellular Assembly and Organization	1.73E-02 - 7.51E-05	144
Cellular Function and Maintenance	1.73E-02 - 7.51E-05	178

Physiological System Development and Function

Name	p-value	#Molecules
Cardiovascular System Development and Function	1.44E-02 - 4.27E-04	33
Organismal Development	1.73E-02 - 4.27E-04	179
Tissue Morphology	1.73E-02 - 4.27E-04	114
Hematological System Development and Function	1.73E-02 - 5.94E-04	90
Immune Cell Trafficking	1.73E-02 - 5.94E-04	33

Top Tox Functions

Assays: Clinical Chemistry and Hematology

Name	p-value	#Molecules
Increased Levels of AST	1.96E-01 - 3.56E-02	2
Increased Levels of Alkaline Phosphatase	3.56E-02 - 3.56E-02	1
Decreased Levels of Albumin	3.76E-01 - 7.00E-02	3
Increased Levels of CRP	7.00E-02 - 7.00E-02	1
Increased Levels of Potassium	7.00E-02 - 7.00E-02	1

Cardiotoxicity

(c) 2000-2017 QIAGEN. All rights reserved.

INGENUITY PATHWAY ANALYSIS

Name	p-value	#Molecules
Congenital Heart Anomaly	5.96E-01 - 2.85E-02	14
Cardiac Dilation	7.00E-02 - 3.49E-02	5
Cardiac Arteriopathy	9.80E-02 - 3.56E-02	4
Pulmonary Hypertension	5.66E-01 - 3.56E-02	3
Cardiac Enlargement	6.25E-01 - 5.63E-02	22

Hepatotoxicity

Name	p-value	#Molecules
Liver Hyperplasia/Hyperproliferation	6.38E-01 - 4.26E-04	277
Hepatocellular Carcinoma	6.38E-01 - 3.56E-02	38
Liver Damage	1.00E00 - 3.56E-02	13
Liver Hematopoiesis	3.04E-01 - 3.56E-02	2
Liver Inflammation/Hepatitis	1.00E00 - 3.56E-02	8

Nephrotoxicity

Name	p-value	#Molecules
Renal Dysfunction	3.56E-02 - 1.37E-03	5
Glomerular Injury	5.38E-01 - 7.25E-03	16
Renal Fibrosis	4.40E-01 - 7.25E-03	4
Renal Inflammation	1.00E00 - 7.58E-03	15
Renal Nephritis	1.00E00 - 7.58E-03	15

Top Networks

ID	Associated Network Functions	Score
1	Cell-To-Cell Signaling and Interaction, Hematological System Development and Function, Immune Cell Trafficking	51

(c) 2000-2017 QIAGEN. All rights reserved.

2	Cell Morphology, Cellular Assembly and Organization, Cellular Function and Maintenance	51
3	Cellular Function and Maintenance, Cellular Movement, Carbohydrate Metabolism	51
4	Organ Morphology, Reproductive System Development and Function, Cancer	46
5	Tissue Morphology, Cellular Assembly and Organization, Cellular Function and Maintenance	44

Top Tox Lists

Name	p-value	Overlap	
Mitochondrial Dysfunction	1.05E-02	7.4 %	13/176
RAR Activation	1.89E-02	6.8 %	13/190
VDR/RXR Activation	5.95E-02	7.7 %	6/78
Increases Liver Damage	9.34E-02	6.2 %	8/130
Cardiac Hypertrophy	9.79E-02	5.0 %	17/338

(c) 2000-2017 QIAGEN. All rights reserved.
